# Identification of an alternative ligand‐binding pocket in peroxisome proliferator‐activated receptor gamma and its correlated selective agonist for promoting beige adipocyte differentiation

**DOI:** 10.1002/mco2.650

**Published:** 2024-07-10

**Authors:** Qiang Tian, Miaohua Wang, Xueting Wang, Zhenli Lei, Owais Ahmad, Dianhua Chen, Wei Zheng, Pingping Shen, Nanfei Yang

**Affiliations:** ^1^ State Key Laboratory of Pharmaceutical Biotechnology, Department of Urology The Affiliated Nanjing Drum Tower Hospital The Affiliated Hospital of Nanjing University Medical School School of Life Sciences Nanjing University Nanjing China; ^2^ Shenzhen Research Institute of Nanjing University Shenzhen China; ^3^ School of Pharmaceutical Sciences Wenzhou Medical University Wenzhou China

## Abstract

The pharmacological activation of peroxisome proliferator‐activated receptor gamma (PPARγ) is a convenient and promising strategy for promoting beige adipocyte biogenesis to combat obesity‐related metabolic disorders. However, thiazolidinediones (TZDs), the full agonists of PPARγ exhibit severe side effects in animal models and in clinical settings. Therefore, the development of efficient and safe PPARγ modulators for the treatment of metabolic diseases is emerging. In this study, using comprehensive methods, we report a previously unidentified ligand‐binding pocket (LBP) in PPARγ and link it to beige adipocyte differentiation. Further virtual screening of 4097 natural compounds based on this novel LBP revealed that saikosaponin A (NJT‐2), a terpenoid compound, can bind to PPARγ to induce coactivator recruitment and effectively activate PPARγ‐mediated transcription of the beige adipocyte program. In a mouse model, NJT‐2 administration efficiently promoted beige adipocyte biogenesis and improved obesity‐associated metabolic dysfunction, with significantly fewer adverse effects than those observed with TZD. Our results not only provide an advanced molecular insight into the structural ligand‐binding details in PPARγ, but also develop a linked selective and safe agonist for obesity treatment.

## Graphical abstract

Identification of an alternative ligand‐binding pocket in proliferator‐activated receptor gamma (PPARγ), and screening out NJT‐2, a natural compound derived from *Bupleurum chinense DC*, binds in this pocket, induces the coactivator recruitment, guides the PPARγ binding to the promotors of beige adipocyte‐related genes, thus enhances white adipose tissue browning and improves the metabolic disorders without the similar adverse effects observed with thiazolidinediones.

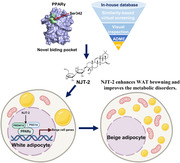

## 1 INTRODUCTION

As a key member of the nuclear receptor family, peroxisome proliferator‐activated receptor gamma (PPARγ) is highly expressed in white adipose tissue (WAT) and is the master regulator of WAT homeostasis.^1^, ^2^ In normal state, PPARγ is indispensable for adipocyte differentiation and WAT development, whereas fat‐specific deletion of PPARγ leads to lipoatrophy and severe metabolic dysfunctions.^3^ In overnutrition state, PPARγ stimulates the expression of genes involved in lipogenesis (synthesis of fatty acids) and adipocyte lipid droplet formation, thereby promoting the uptake of fatty acids and their conversion into triglycerides for storage within adipocytes in WAT.^4^ Simultaneously, PPARγ enhances insulin‐sensitivity gene expressions such as *Glut4*, to facilitate glucose uptake and metabolism, thereby maintaining glucose homeostasis.^5^ Furthermore, PPARγ activation can trigger an interesting cell biological process in WAT, in which this nuclear receptor full agonism induces the beige adipocyte biogenesis.^6^ In this situation, PPARγ transactivates the mitochondrial respiration and thermogenic genes, such as *Ucp1*, *Cox8b* and *Plin5*, which further supports the thermogenesis and energy expenditure in WAT. Therefore, pharmacological PPARγ agonism is considered a promising strategy for the treatment of metabolic diseases.

Although several natural lipids and their derivatives can activate PPARγ, till date, the complete endogenous PPARγ ligands have not been identified.^7^ In contrast, thiazolidinediones (TZDs), the synthetic full agonists of PPARγ with robust insulin‐sensitizing functions, have been used as first‐line drugs in the clinical treatment of type 2 diabetes.^8^ However, long‐term treatment with TZDs causes several severe side effects, such as weight gain, fluid retention, osteoporosis, and cardiovascular disorders.^9^ Due to cardiovascular side effects, the US Food and Drug Administration (FDA) and European authorities provisionally retracted the approval of rosiglitazone (RSG)^10^, ^11^; however, in 2013, after a series of clinical studies, the FDA abandoned this decision. Nevertheless, glitazone treatment still needs to be approved during retraction.^12^ Therefore, the adverse effects of TZDs must be considered. However, the development of novel, effective, and safer PPARγ ligands is emerging. A class of “selective PPARγ modulators” (SPPARγMs), which act as partial agonists to specifically modulate the trans activity of PPARγ, has been reported.^13^ SPPARγMs have been developed to optimize gene‐expression signatures to generate metabolically beneficial effects while minimizing side effects.

Multiple attempts to develop SPPARγMs resulted in the creation of various PPARγ ligands. For example, amorfrutins, a class of 2‐hydroxy benzoic acid derivatives, exerts mild PPARγ‐activation activity and significant insulin‐sensitizing effect in animal models and do not exhibit side effects with TZDs.^14^, ^15^ Moreover, telmisartan, an angiotensin receptor inhibitor, binds to PPARγ as a SPPARγM.^16^ In contrast to TZDs, telmisartan induces the destabilization of helix 12 and stabilizes helix H3, producing a distinct conformational change. Telmisartan has been evaluated in clinical trials for the treatment of various diseases.^17^, ^18^ Ongoing basic research on the post‐translational modifications (PTMs) of PPARγ provides new opportunities for developing more efficient SPPARγMs. Choi et al. reported that cyclin‐dependent kinase 5 (CDK5)‐mediated serine 273 phosphorylation inactivates PPARγ and inhibits the expression of insulin‐sensitive genes.^19^ Small molecules MRL24 and SR1664 can block the phosphorylation of PPARγ at serine 273, selectively modulate the PPARγ transactivation to insulin‐sensitivity genes, and improve type 2 diabetes.^20^, ^21^ These compounds have fewer adverse effects on TZDs, and SR1664 has been tested in clinical trials.^22^, ^23^ Recently, our study identified PPARγ T166 phosphorylation and found that this PTM in PPARγ DNA‐binding domain (DBD) switched the white and beige adipocyte transition.^24^ Our compound‐screening assay uncovered a SPPARγM, 2‐cyano‐3,12‐dioxo‐olean‐1,9‐dien‐28‐oic acid (CDDO), that specifically inhibited T166 phosphorylation and enhanced the beige‐adipocyte differentiation. Structural biology strategy provides the evidence that CDDO binding induced the conformational changes of PPARγ, which differs from that by TZD, suggesting that the binding position and characteristics of CDDO are unique. Identifying the CDDO‐binding pocket and discovering key residues in PPARγ are crucial for understanding the CDDO‐activated PPARγ transcriptional actions, and this might be a convenient way to develop new SPPARγMs.

Here, by comprehensive use of structural modeling and biochemical approaches, we dissected the binding characteristics of CDDO in PPARγ, discovered the correlated key residue in PPARγ ligand‐binding domain (LBD), and successfully identified an uncharacterized small molecule‐binding pocket in PPARγ. Based on the novel binding pocket, a library of 4097 natural compounds was constructed, and two‐dimensional (2D) and three‐dimensional (3D) similarity‐based virtual screenings were performed. These series of studies revealed an active molecule, NJT‐2, a terpenoid compound, which was able to bind in the novel pocket and activate PPARγ. The TZD contacted the helix H12 of PPARγ, stabilizing the agonist conformation through a direct hydrogen bond to Tyr473, His323, or His449. In contrast to the bonding mechanism of TZD to PPARγ, NJT‐2 directly bonded by hydrogen bonding with Arg280, Glu259, Ser342, Glu342, Glu291, and Glu295 on the helixes H3 and H2ʹ. In addition, PPARγ had higher binding affinity to NJT‐2 than to CDDO, resulting in the promotion of beige adipocyte differentiation in vitro compared to CDDO. Importantly, the pharmacological efficacy evaluation indicated that NJT‐2 exhibited high activity in activating beige adipocyte differentiation and effectively improved obesity‐related metabolic disorders, while without causing the adverse effects that induced by TZD. These advantages in molecular binding, pharmacological effects, and low side effects collectively support the application prospects of NJT‐2 as a novel PPARγ agonist.

## 2 RESULTS

### 2.1 Identification of a novel small molecule‐binding pocket in PPARγ LBD

In our previous study, we found that CDDO, a partial agonist of PPARγ, possessed high efficiency in inducing WAT browning. Differential hydrogen deuterium exchange (HDX) revealed that CDDO bond to the PPARγ LBD, caused a large magnitude change in DBD, and inhibited protein kinase C alpha (PKCα)‐mediated phosphorylation at the T166 site of DBD. This effect led to the modulation of PPARγ  conformation and changed its transcriptional action, which enhanced the beige cell differentiation. However, RSG, the full agonist of PPARγ had limited effect on the DBD conformation.^24^ This evidence proved that the binding characteristics of CDDO differed from those of RSG, and we considered that CDDO was able to bind to a novel pocket in the PPARγ LBD. Thus, identifying the binding position of CDDO in PPARγ is useful for developing new PPARγ selective ligand.

**FIGURE 1:**
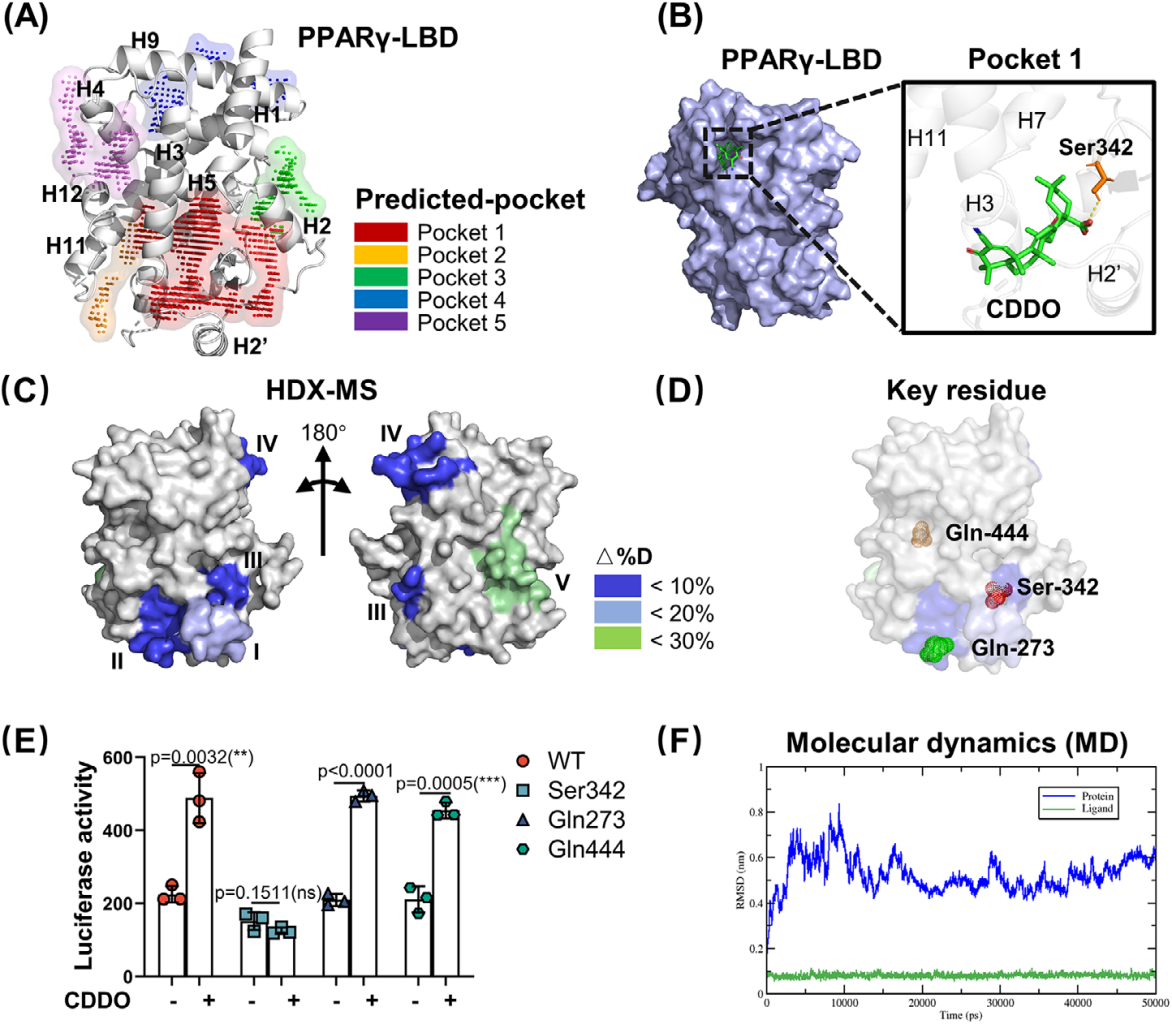
Identification of a novel 2‐cyano‐3,12‐dioxo‐olean‐1,9‐dien‐28‐oic acid (CDDO) binding pocket in proliferator‐activated receptor gamma (PPARγ). (A) SiteMap prediction of five high‐rank possible binding pockets in PPARγ ligand‐binding domain (LBD). (B) The predicted pocket 1 that CDDO binding and the interacted Ser342 residue. (C) Re‐analyzing the published hydrogen deuterium exchange (HDX)‐MS data and mapping five dramatical exchange regions induced by CDDO (regions I‒V). (D) The key residues in predicted CDDO‒PPARγ complex: Ser342 (pocket 1), Gln273 (pocket 2), and Gln444 (pocket 4). (E) Luciferase reporter assay evaluating the transcriptional activity of wild type (WT) and site mutated PPARγ with or without CDDO treatment in 293T cells. Every point represented one experimental replication (*n* = 3). (F) Changes in root mean square deviation (RMSD) values of the CDDO‒PPARγ complex within Ser342 as the binding pocket. Data are expressed as the mean ± standard deviation (SD). Data are analyzed by unpaired Student's *t*‐test. ^*^ *p* < 0.05, ^**^ *p* < 0.01, ^***^ *p* < 0.001.

To achieve this goal, we first applied SiteMap, a Maestro plug‐in, to map the potential binding pockets for small molecules in PPARγ LBD (PDB:3E00), and top five binding pockets were successfully predicted (marked by numbers 1‒5 and shown in Figure 1A). Table S1 summarizes the properties of the pockets with the site and D scores. In these five pockets, pocket 1, with high site and D scores, occupied a large fraction of H2, H3, and H5. To analyze the possible binding positions of CDDO in these five pockets, we used the Glide program for molecular docking. The docking positions are shown in Figures 1B and S1A‒D. In the pocket 1, CDDO was located at a deep cavity with a high docking score of ‒4.196 (Table S1), and the C28 carboxy group of CDDO formed a hydrogen bond with the Ser342 residue in the H2ʹ region (Figure 1B). In the pocket 2, the C28 carboxy group formed a hydrogen bonding interaction with Gln273 in H3 region, and docking score was ‒2.143 (Figure S1A). In pocket 3, the C3 carbonyl group of CDDO formed a hydrogen bond with LYS‐336 on the surface of H5, and the C12 carbonyl group formed a hydrogen bond with Lys373 on the surface of the H6 region, resulting in a docking score of ‒2.650 (Figure S1B). In pocket 4, the C28 carboxy and C12 carbonyl groups of CDDO formed hydrogen bonds with Gln444 and Lys438 in the H11 region, respectively (docking score, ‒2.531) (Figure S1C). In pocket 5, the C28 carboxy group of CDDO formed hydrogen bonds with Lys301 in the H3 region, and the C3 carbonyl group formed hydrogen bonds with Lys319 in the H4 region (docking score, ‒2.531) (Figure S1D). Combining these results, pocket 1 was most probably the best region for CDDO binding to the PPARγ LBD.

Based on the above prediction, to further investigate and confirm the binding pocket, we re‐analyzed the differential HDX data obtained in our recent study^24^ and constructed a virtual structure model of the LBD using PyMol. As shown in Figure S1E, CDDO binding induced a distinct HDX profile in the LBD compared with that in the RSG. In RSG–PPARγ complex, RSG induced significant HDX change ratio in helices 5, 11, and 12, while CDDO led to significant HDX change ratio in helices 2′, 3, 5, 9, and 10. Furthermore, we summarized five dramatical exchange regions that were induced by CDDO in PPARγ LBD (Figure 1C). Region I, covering residues 256‒273, showed an average decrease of 20% in deuterium uptake. Regions II (residues 275‒285), III (residues 335‒348), and IV (residues 421‒431) showed approximately 10% decrease in deuterium uptake. Region V (residues 440‒453) exhibited approximately 30% increase in deuterium uptake. Collectively, the HDX data suggested that these five regions are possible binding pockets for CDDO. On comparing SiteMap prediction and HDX assay data, we found that the pockets 1, 2, and 4 covered regions III, I, and V, respectively. Region III contained a deep hydrophobic pocket, indicating that CDDO could be located in this cavity. These results suggest that region III is a suitable pocket for CDDO binding to PPARγ LBD. Based on the results of molecular docking and HDX‐MS, we selected Ser342 (pocket 1), Gln273 (pocket 2), and Gln444 (pocket 4) as key residues for further study (Figure 1D). Thus, the candidate binding sites for the CDDO–PPARγ complexes were most likely to be Ser342(S342), Gln273(Q273), or Gln444(Q444).

To verify this speculation, we used a luciferase reporter assay with alanine point mutant PPARγ proteins (S342A, Q273A, and Q444A). As shown in Figure 1E, only the S342A mutant totally blocked the CDDO‐induced transcriptional activation of PPARγ. This indicates that the S342 residue is the critical site for CDDO binding, and the corresponding pocket 1 is the binding region for CDDO. Molecular dynamics (MD) simulations were performed to explore the stability of the PPARγ–CDDO complex within the Ser342 binding site (pocket 1). The root mean square deviation (RMSD) values showed that the model was stable in the last 20 ns, fluctuating less than 0.3 Å (Figure 1F) and forming four hydrogen bonds (Figure S1F). Combining these results shows that Ser342 contributes to the CDDO–PPARγ interaction. The pocket 1 with a high druggable score has maintained the stability of the CDDO–PPARγ complex, suggesting that pocket 1 and Ser342 site in it were good candidates for further virtual screening.

### 2.2 2D and 3D similarity‐based virtual screening

**FIGURE 2:**
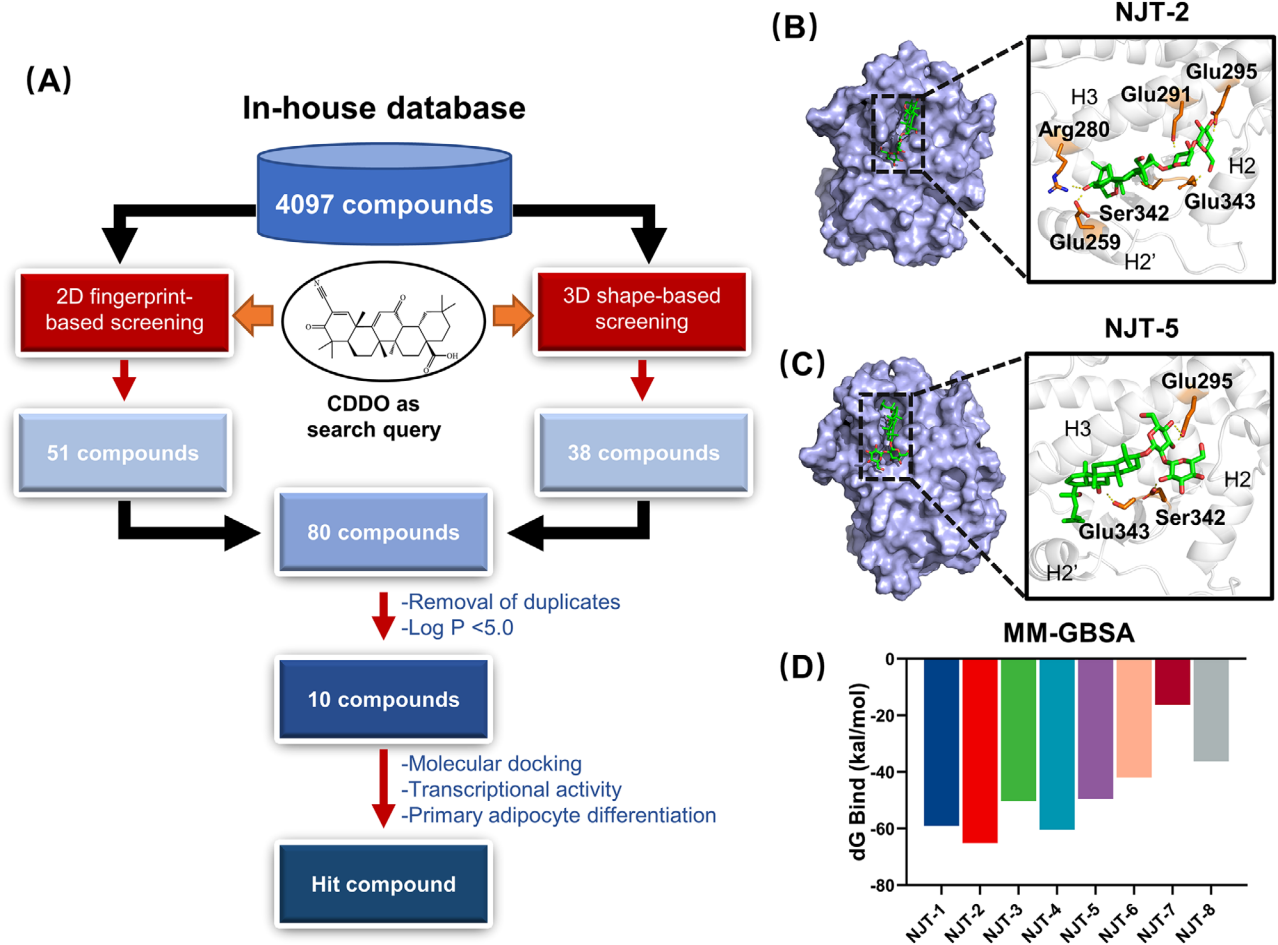
Virtual screening identifies NJT‐2 and NJT‐5 bind in pocket 1. (A) The workflow of two‐dimensional (2D) and three‐dimensional (3D) similarity‐based virtual screening based on in‐house natural compound library. (B and C) The docking model of NJT‐2 (B) or NJT‐5 (C) with pocket 1 in proliferator‐activated receptor gamma (PPARγ) ligand‐binding domain (LBD). (D) Generalized Born and surface area solvation (MM‐GBSA) analysis of the binding free energies of the compound to PPARγ.

Virtual screening is widely used in drug discovery. In this study, 2D and 3D similarity‐based virtual screening was used to identify CDDO‐like compounds. First, we created an in‐house chemical library derived from traditional Chinese medicines (TCMs). The compounds in this library are derived from 91 Chinese herbal medicines recorded in ancient Chinese medical texts (including Shang Han Lun, Jin Kui Yao Lve, and Pi Wei Lun) for their therapeutic effects on metabolic diseases. As shown in Figure S2A, some herbs appeared in all three ancient Chinese medical texts. By comprehensively reviewing the formulas and the cited herbs in the three ancient texts, integrating annotations from the Traditional Chinese Medicine Systems Pharmacology Database and Analysis Platform (TCMSP), Traditional Chinese Medicines Integrated Database (TCMID), and CSCB online databases, 4097 natural compounds were collected and recorded in our virtual in‐house small molecule library (Figure S2B) (refer Section 4). Then, using molecular access system (MACCS), extended‐connectivity fingerprints (ECFP), and topological 2D fingerprints, we screened 51 CDDO‐like compounds (top 20). Thirty‐eight compounds (top 20 ranked) were selected based on 3D shape fingerprints. After the removal of duplicates, 80 compounds were identified. For better physicochemical properties and structural diversity, 10 compounds were further selected according to the value of log *p *< 5.0 and cluster analysis (Figure 2A and Table S2), including saikosaponin D (NJT‐1), saikosaponin A (NJT‐2), licorice‐saponin F3 (NJT‐3), saikosaponin B3 (NJT‐4), ginsenoside Rg3 (NJT‐5), longispinogenin 3‐O‐beta‐D‐glucuronopyranoside (NJT‐6), uralsaponin B (NJT‐7), 26‐deoxyactein (NJT‐8), arjungenin (NJT‐9), and hederagenin 3‐O‐arabinoside (NJT‐10).

To discover the novel selective modulators of PPARγ in these 10 compounds, we applied the molecular docking within identified pocket 1. The docking scores are shown in Table S2. The docking results showed that the high docking scores of NJT‐1 and NJT‐2 compounds were 5.89 and 5.71, respectively. Detailed docking model revealed that NJT‐1 formed hydrogen‐bonding interactions with Arg280, Glu259, Glu291, and Glu295 with PPARγ (Figure S2C). NJT‐2 formed six hydrogen bonds with Arg280, Glu259, Ser342, Glu342, Glu291, and Glu295 (Figure 2B). NJT‐1 and NJT‐2 are a pair of isomers, and their structural difference is in the configuration of the hydroxyl group at C16.^25^ However, NJT‐1 is highly toxic, causing hepatotoxicity, neurotoxicity, hemolysis, and cardiotoxicity.^26^ NJT‐3 and NJT‐4 could also target PPARγ with the docking scores of 5.49 and 4.96, respectively. However, NJT‐3 and NJT‐4 did not interact with Ser342 (Figure S2D,E). NJT‐5 formed hydrogen bonds with Ser342, Glu‐343, and Glu‐295 (Figure 2C) with the docking score was 4.53. NJT‐6, and NJT‐7 and NJT‐8 had lower affinity to PPARγ with the docking scores were 4.18, 4.12, and 3.11, respectively, and all of them exhibited the interaction with Ser342 (Figure S2F‒H). At last, NJT‐9 and NJT‐10 could not be docked in pocket. Furthermore, the generalized Born and surface area solvation (MM‐GBSA) method was used to determine the binding free energies of the compound‒PPARγ complexes. MM‐GBSA results revealed that NJT‐2, NJT‐4, and NJT‐1 provided the higher binding affinity of PPARγ with Δ*G* bind values of ‒65.11, ‒60.46, and ‒59.06 kcal/mol, respectively (Figure 2D). Therefore, based on their binding scores and interactions with Ser342, we selected NJT‐2 and NJT‐5 for further biological evaluation.

### 2.3 NJT‐2‐activated PPARγ and promoted beige adipocyte differentiation in vitro

To further study the biological activity of NJT‐2 and NJT‐5 on PPARγ and the correlated function in adipocytes, we first conducted the cell viability assay on primary adipose‐derived stem cell (ADSC) or 3T3‐L1 cell line. As shown in Figure S3A,B, cell counting kit‐8 (CCK‐8) assay indicated that NJT‐2 at concentrations below 10 µM does not induce significant cytotoxicity in either ADSC or 3T3‐L1 cells. The Annexin V/propidium iodide (PI) apoptosis kit evaluation confirmed the results obtained by the CCK‐8 assay (Figure S3C,D). Considering the differentiated mature adipocytes could not be analyzed by flow cytometry, we combined used TUNEL and CCK8 assay to evaluated the cytotoxicity of NJT‐2 on adipocytes. The results showed that NJT‐2 at concentrations below 1 µM were safe for mature adipocytes (Figure S3E,F). Parallelly, we also assessed the cytotoxicity of NJT‐5, and the data suggested that NJT‐5 at 20 µM did not induce significant cytotoxicity (Figure S3A,B,E).

We compared the bioactivity of NJT‐2 and NJT‐5. To allow for comparison with equal concentrations of RSG or CDDO (positive controls) in subsequent experiments, we chose to use a dose of 200 nM NJT‐2 and NJT‐5 for in vitro activity assessment. First, in the primary adipocyte differentiation model, both NJT‐2 and NJT‐5 promoted de novo adipogenesis (Figure S4A,B), and NJT‐2 exhibited a stronger capacity to induce differentiation. Second, compared to NJT‐5, NJT‐2 significantly promoted the expression of adipogenic genes, such as *Fabp4*, *Fasn*, and *CD36* (Figure S4C). These data suggested that the bioactivity of NJT‐2 on adipocyte‐differentiation promotion was higher than that of NJT‐5. Therefore, we selected NJT‐2 cells for further evaluation of beige cell biogenesis and metabolic regulation.

**FIGURE 3:**
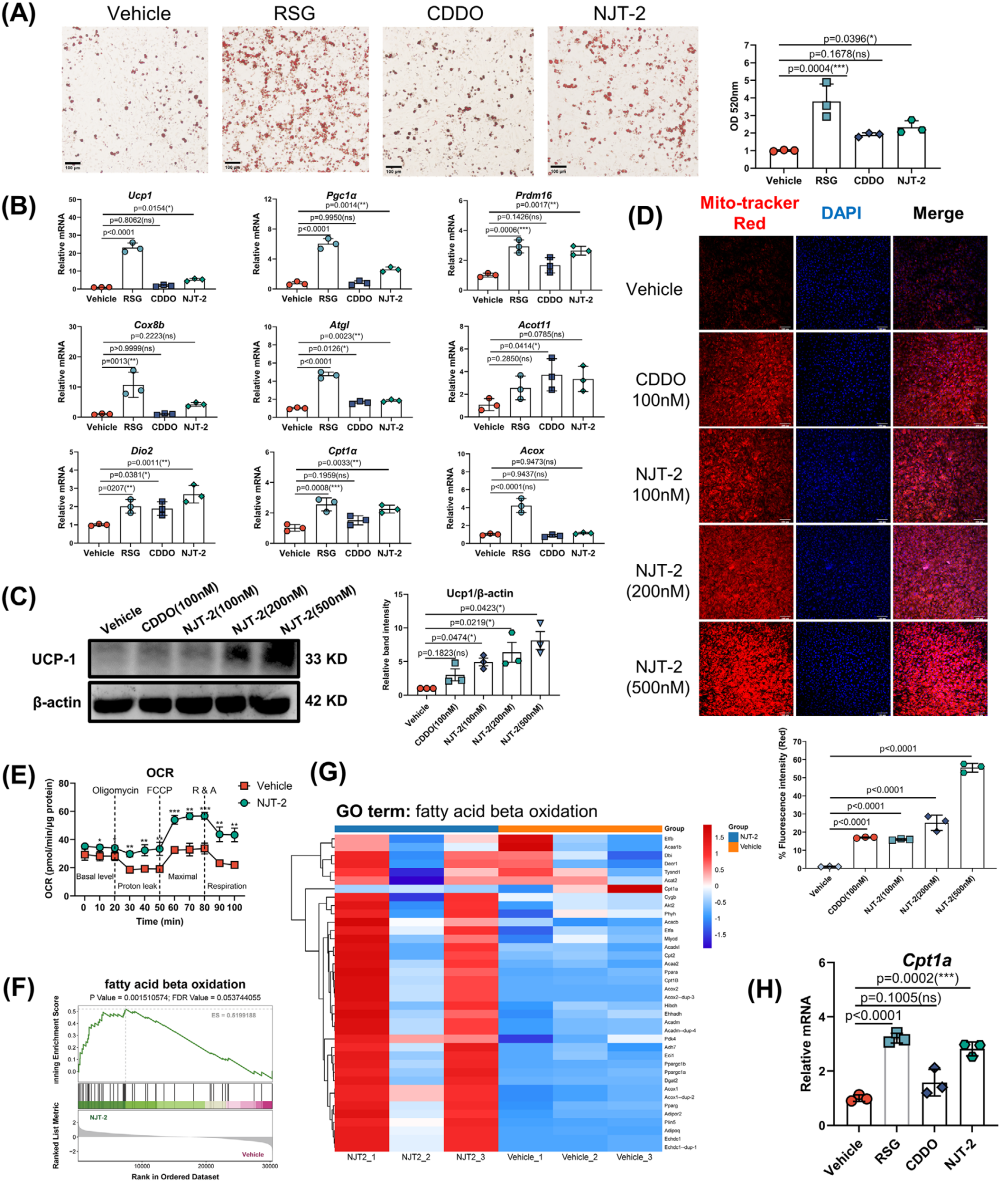
NJT‐2 effectively promotes the beige adipocyte differentiation in vitro. (A) The adipose‐derived stem cell (ADSC) cells were isolated from subcutaneous adipose tissue (SAT) and were differentiated into mature adipocytes with or without the compound treatment. The concentration of compounds (rosiglitazone [RSG], 2‐cyano‐3,12‐dioxo‐olean‐1,9‐dien‐28‐oic acid [CDDO], and NJT‐2) was 200 nM. Oil red O staining and quantitative analysis of the content of lipid droplets. In statistical chart, every point represented one experimental replication (*n* = 3). (B) Beige cell‐related marker gene expressions were evaluated by qPCR. Every point represented one experimental replication (*n* = 3). (C) Western blotting analysis of UCP1 protein content in cultural differentiated adipocytes. This experiment has been repeated three times and every point in statistical chart represents one experimental replication (*n* = 3). (D) Confocal microscopy analysis of the mitochondrial potential (MitoTracker red) in differentiated adipocytes. 100× magnification; scale bar, 100 µm. This experiment has been repeated three times and every point in statistical chart represents one experimental replication (*n* = 3). (E) Oxygen consumption rate (OCR) of adipocyte mitochondria (*n* = 5). (F) Gene set enrichment analysis (GSEA) analysis of differentially expressed genes in RNA‐sequencing (RNA‐seq). (G) Expression of fatty acid beta oxidation genes by RNA‐seq (*n* = 3). *p* < 0.05 according to an unpaired two‐sided Student's *t*‐test and **|**log2 (fold change [FC])**|** > 1. (H) qPCR analysis of Cpt1a mRNA level in cultural differentiated adipocytes. Every point represented one experimental replication (*n* = 3). Data are expressed as the mean ± standard deviation (SD). Data are analyzed by one‐way analysis of variance (ANOVA) followed by Dunnett's test (A‒D, H) or two‐way ANOVA followed by Bonferroni's test (E). ^*^ *p* < 0.05, ^**^ *p* < 0.01, ^***^ *p* < 0.001.

As NJT‐2 effectively promotes the differentiation of ADSC cells into adipocytes (Figure 3A), it also significantly enhanced the expression levels of beige adipocyte‐related genes, including *Ucp1*, *Prdm16*, *Cox8b*, *Dio2*, *Atgl*, *Acot11*, etc. (Figure 3B). As an important marker of beige adipocytes,^27^ the level of uncoupling protein‐1 (UCP1) was parallelly detected by Western Blotting. These results suggest that NJT‐2 increased the protein level of UCP1 in a dose‐dependent manner (Figure 3C). Since beige adipocyte biogenesis is tightly linked to mitochondrial activity, we analyzed the mitochondrial membrane potential using Mitotracker Red. As shown in Figure 3D, NJT‐2 enhanced the mitochondrial activity in adipocytes. Subsequent bioenergetic measurements confirmed that NJT‐2 induced a marked increase in the oxygen consumption rate (OCR) (Figure 3E), indicating that this small molecule supports energy expenditure in beige adipocytes.

Considering that beige adipocytes possess enhanced glucose uptake function, we quantified the glucose content in the culture media and calculated glucose consumption upon NJT‐2 treatment. The results showed that similar to the efficacy of RSG, NJT‐2 treatment significantly increased glucose uptake by adipocytes (Figure S5A). We analyzed the gene expression of *Glut4*, the major glucose transporter isoform in adipocytes and found that its mRNA level was dramatically increased by NJT‐2 administration (Figure S5B), which supported the conclusion obtained by glucose quantification that NJT‐2 could upregulate *Glut4* expression and promote glucose uptake. We next analyzed the effect of NJT‐2 on lipid metabolism. Previous studies indicated that lipid catabolism and anabolism are inseparable in thermogenic adipocytes. Brown and beige adipocytes upregulate the fatty acid synthesis pathway to support mitochondria‐mediated fatty acid oxidation (FAO) and heat dissipation. Here, we quantified the fatty acid content and relative gene expression of two rate‐limiting enzymes (*Acc1* and *Fasn*) in adipocyte fatty acid synthesis. We found that NJT‐2 treatment markedly increased fatty acid levels and significantly enhanced *Acc1* and *Fasn* mRNA levels (Figure S5C,D), indicating that NJT‐2 enhanced fatty acid synthesis. To further confirm our observations, we performed unbiased RNA‐sequencing (RNA‐seq) to describe the metabolic phenotype of NJT‐2‐treated adipocytes. The results showed that the vehicle and NJT‐2‐treated adipocytes had distinct molecular signatures (Figure S5E). Gene set enrichment analysis (GSEA) indicated that NJT‐2 regulates both lipid biosynthesis and FAO (Figures S5F and 3F). Regarding the lipid biosynthesis process, NJT‐2 treatment enriched a unique profile of gene sets, including *Fasn*, *Cd36*, *Fabp4*, and *Scd1/2* (Figure S5G). On the other hand, for FAO catabolism process, NJT‐2 significantly increased fatty acid oxidization gene expressions, such as *Cpt1b*, *Plin5*, *Dgat2*, *Acox*, *Ppargc1a*, etc. (Figure 3F,G). Quantitative real‐time polymerase chain reaction (qPCR) confirmed that *Cpt1b*, the rate‐limiting enzyme in FAO, was highly induced by NJT‐2 treatment (Figure 3H). Meanwhile, the several glucose metabolism genes related to insulin sensitivity were also significantly induced by NJT‐2, including *Glut4*, *Pparα*, and *Adipoq* (Figure S5H‒I). Collectively, all these data suggest that NJT‐2 remodels glucose and lipid metabolisms during the beige adipocyte differentiation.

### 2.4 NJT‐2 modulates the transcriptional action of PPARγ

**FIGURE 4:**
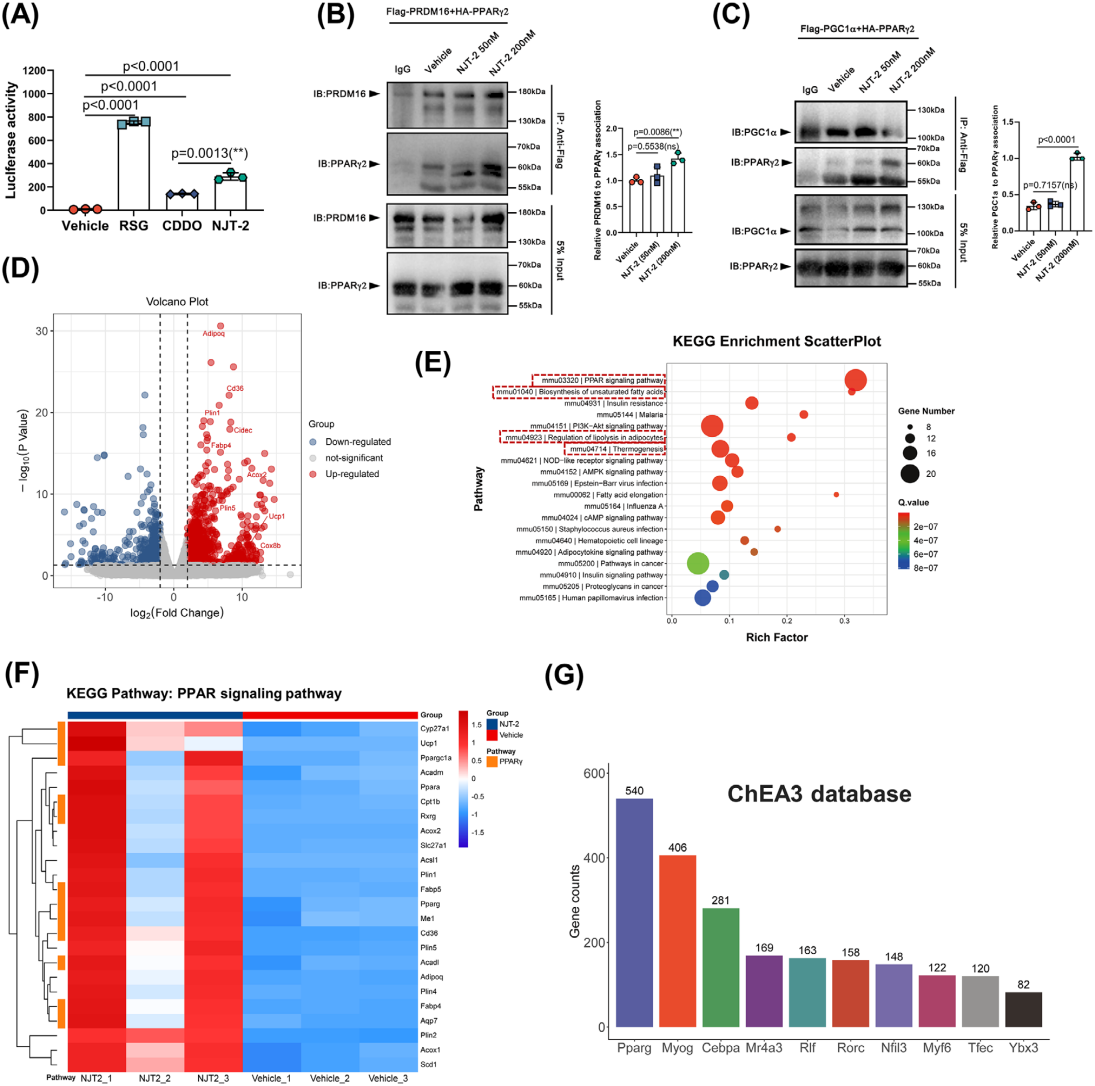
NJT‐2 modulates the transcriptional action of proliferator‐activated receptor gamma (PPARγ). (A) Luciferase reporter assay evaluating the transcriptional activity of wild‐type PPARγ upon the treatment with 200 nM rosiglitazone (RSG), 2‐cyano‐3,12‐dioxo‐olean‐1,9‐dien‐28‐oic acid (CDDO), or NJT‐2. Every point represented one experimental replication (*n* = 3). (B and C) Co‐IP analysis of the interaction between PPARγ and PRDM16 (B) or PGC11α (C) in the 293T cells. Experiments were repeated three times. Every point in statistical chart represented one experimental replication (*n* = 3). (D) RNA‐sequencing (RNA‐seq) was performed in vehicle or NJT‐2‐treated differentiated adipocyte (*n* = 3). Volcano plot showing beige cell markers. (E) Kyoto Encyclopedia of Genes and Genomes (KEGG) analysis of differentially expressed genes between vehicle and NJT‐2 group. (F) Heatmap of differentially expressed genes related to PPAR signaling pathway. (G) ChIP‐X enrichment analysis (ChEA) cluster of the upstream transcriptional factors controlling differentially expressed genes. Data are expressed as the mean ± standard deviation (SD). Data are analyzed by one‐way analysis of variance (ANOVA) followed by Dunnett's test (A‒C). ^*^ *p* < 0.05, ^**^ *p* < 0.01, ^***^ *p* < 0.001.

To examine the mechanism by which NJT‐2‐activated PPARγ and promoted beige‐adipocyte differentiation, we evaluated the effects of this small molecule on PPARγ transcriptional action. First, to prove NJT‐2 could bind to PPARγ in mammalian cells, we fused green fluorescent protein (GFP) PPARγ LBD and overexpressed the fusion protein in 293T cells. GFP overexpression was used as a control. Then, microscale thermophoresis (MST) was applied to evaluate the affinity of NJT‐2 to PPARγ LBD in cells. The results showed that NJT‐2 could bind to the LBD of PPARγ with a *K*_d_ value of 5.07 µM in mammalian cells (Figure S6A). Second, in luciferase reporter system, we observed that NJT‐2 significantly enhanced the transcriptional activity of PPARγ, and compared to that of CDDO, NJT‐2 exhibited stronger capacity to activate PPARγ. Compared to the complete agonist RSG, NJT‐2 achieved approximately 40% transactivation activity, indicating that NJT‐2 is a partial agonist of PPARγ (Figure 4A). Subsequently, transcriptional cofactor binding was analyzed. PRDM16 and PGC1α are defined as the pro‐beiging co‐factors that bind to PPARγ and guide it to promote the beige cell‐related gene transcription.^28^, ^29^ Here, we used co‐immunoprecipitation (Co‐IP) to evaluate the interactions of PPARγ and PRDM16 or PGC1α. As showed in Figure 4B,C, 200 nM NJT‐2 treatment significantly enhanced the interplay between PPARγ and PRDM16 or PGC1α, which suggested that NJT‐2 possessed the capacity to facilitate the pro‐beiging transcriptional complex assembly. These results indicated that NJT‐2 can directly bind to PPARγ, induce co‐factor interaction, and activate the transcriptional PPARγ activity.

To further verify the effect of NJT‐2 on PPARγ pathway and associated influence on beige‐adipocyte profile, we re‐analyzed the bulk RNA‐seq data. The volcano plot of the differentially expressed genes indicated that NJT‐2 treatment markedly induced the expression of beige adipocyte‐related genes such as *Ucp1*, *Cidec*, *Acox2*, *Plin5*, and *Cox8b* (Figure 4D). Gene Ontology (GO) enrichment revealed that NJT‐2 regulates brown fat differentiation and lipid metabolism (Figure S6B). For the genes enriched in the brown‐fat differentiation process, besides *Ucp1*, we observed several upregulated genes related to the beige program, such as *Ppargc1a* (encode PGC1α), *Scd1* (catalyze the synthesis of monounsaturated fatty acids), and *Mrap* (a key regulator in adrenergic signaling) (Figure S6C). Kyoto Encyclopedia of Genes and Genomes (KEGG) enrichment analysis provided evidence that the most significant NJT‐2‐regulated process was the PPAR‐signaling pathway (Figure 4E). In this pathway, we clustered the PPARγ downstream genes and compared the expression levels between vehicle and NJT‐2 group. As shown in the heatmap in Figure 4F, the orange modules marked PPARγ transcription genes, such as *Cyp27a1*, *Ucp1*, *Ppargc1a*, *Cpt1b*, and *Acadl*, which were highly linked to beige cell function. To further confirm the role of NJT‐2 in regulating PPARγ pathway, the ChIP‐X enrichment analysis (ChEA; https://maayanlab.cloud/chea3/) was used to select the PPARγ target genes. In contrast to KEGG, ChEA‐enriched PPARγ‐transcribed genes were based on real ChIP‐seq databases. We found that 540 genes were identified as the NJT‐2 induced the PPARγ direct‐binding genes (Figure 4G and Dataset S1). Among beige adipocyte markers in these 540 genes, we found that the *Ucp1* and *Cidec* exhibited the most dramatic upregulation (log fold change [FC] > 8) under NJT‐2 treatment (Figure S6D and Dataset S1). Moreover, these two genes were the direct downstream targets of PPARγ. Furthermore, to verify the bioinformatics analysis, cultured adipocytes were subjected to ChIP‐qPCR. As expected, PPARγ preferred to bind the promoters/enhancers of *Ucp1* and *Cidec* genes under the treatment of NJT‐2 (Figure S6E). Considered together, all these data indicate that NJT‐2 directly targets PPARγ and regulates the transcriptional action of PPARγ on beige cell genes.

### 2.5 NJT‐2‐induced WAT browning in vivo

Following the examination of cellular characteristics and molecular processes, the pro‐beiging activity of NJT‐2 was further confirmed in an animal model. Prior to initiating pharmacological investigations, drug toxicity assays were performed. C57BL/6J mice were administered five different doses of the drug once daily (10, 20, 50, and 100 mg/kg) for a duration of 2 weeks. Body mass assessment indicated that NJT‐2 did not induce notable alterations in the mice (Figure S7A). Analysis of alanine aminotransferase (ALT) and aspartate aminotransferase (AST) levels in the blood as indicators of liver damage demonstrated that even after high‐dose administration of NJT‐2 (100 mg/kg), AST and ALT concentrations did not increase (Figure S7B). Moreover, the levels of creatinine and blood urea nitrogen, two markers of kidney injury, were not influenced by the administration of NJT‐2 (Figure S7C). Tissue samples from key organs of mice treated with NJT‐2 were examined, followed by visceral index (percentage to body weight) and histopathological assessment, which revealed that NJT‐2 did not cause significant damage to the organs (Figure S7D,E). In summary, in vivo toxicity assessments suggested that NJT‐2 displayed minimal toxicity and was characterized by its safety profile.

**FIGURE 5:**
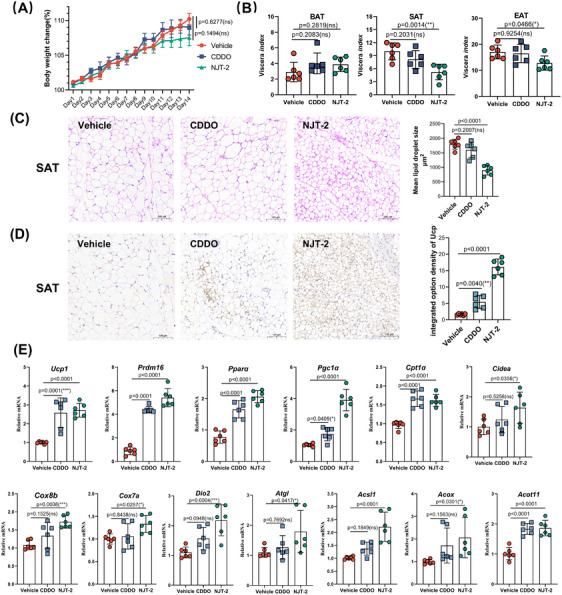
NJT‐2 induces the beige adipocyte biogenesis in vivo. (A) Body weight curve of 6‐week‐old C57/B6J mice treated with vehicle, 2‐cyano‐3,12‐dioxo‐olean‐1,9‐dien‐28‐oic acid (CDDO) (10 mg/kg), or NJT‐2 (10 mg/kg) for 14 days (*n* = 6). (B) The viscera index of adipose tissues (percentage to body weight) from mice in the three groups (*n* = 6). (C) Hematoxylin‒eosin (H&E) staining of subcutaneous adipose tissue (SAT). 100× magnification, scale bar, 100 µm (*n* = 6). (D) Uncoupling protein‐1 (UCP1) immunohistochemical staining of SAT. 100× magnification, scale bar, 100 µm (*n* = 6). (E) Relative mRNA levels of browning‐related genes in SAT (*n* = 6). Data are expressed as the mean ± standard deviation (SD). Data are analyzed by one‐way analysis of variance (ANOVA) followed by Dunnett's test (B‒E) or two‐way ANOVA followed by Bonferroni's test (A). ^*^ *p* < 0.05, ^**^ *p* < 0.01, ^***^ *p* < 0.001.

The mice were treated with NJT‐2 at a dose of 10 mg/kg to assess its effect on adipose browning. CDDO was used as the positive control. Over the 14‐day treatment period, the NJT‐2 group did not exhibit significant change in body weight compared with the vehicle group (Figure 5A). The adipose mass evaluation suggested that NJT‐2 decreased WAT mass (subcutaneous adipose tissue [SAT] and epididymal adipose tissue [EAT]), while had no effect of brown adipose tissue (BAT) mass (Figure 5B). By analyzing the histological morphology of SAT, we found that NJT‐2 demonstrated a higher capacity than CDDO to promote the generation of multilocular adipocytes (Figure 5C). Analysis of the UCP1 positive area in the SAT revealed that NJT‐2 exhibited approximately 2‒3 times higher efficacy in stimulating UCP1 expression (Figure 5D). At the mRNA level, NJT‐2 significantly increased the expression of beige adipocyte‐related genes such as *Ucp1*, *Prdm16*, *Pgc1a*, and *Cox8b* (Figure 5E). Collectively, these data demonstrated that NJT‐2 safely and effectively promotes WAT browning.

### 2.6 NJT‐2 alleviated HFD‐induced obesity by promoting WAT browning

**FIGURE 6:**
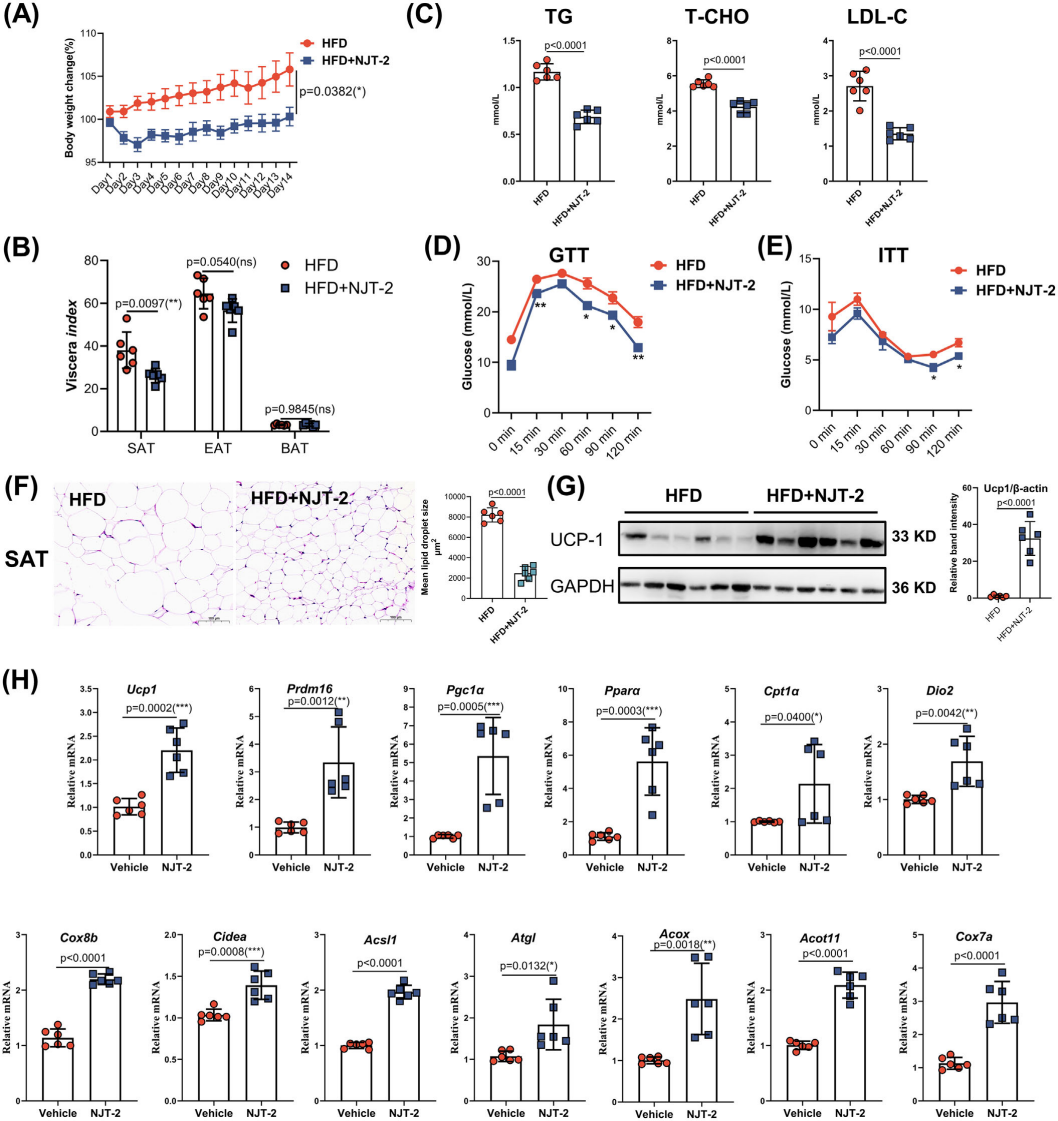
NJT‐2 attenuates the metabolic disorders in diet‐induced obese mice. (A) Body weight curve of high‐fat diet (HFD) C57/B6J mice treated with (10 mg/kg) for 14 days (*n* = 6). (B) The viscera index of adipose tissues (percentage to body weight) from mice in the three groups (*n* = 6). (C) The concentrations of triacylglycerols (TG), total cholesterol (T‐CHO), and low‐density lipoprotein cholesterol (LDL‐C) were measured by commercial kits (*n* = 6). (D and E) Glucose tolerance test (GTT) (D) and insulin tolerance test (ITT) (E) in vehicle or NJT‐2‐treated HFD obese mice (*n* = 6). (F) Hematoxylin‒eosin (H&E) staining of subcutaneous adipose tissue (SAT). 100× magnification, scale bar, 100 µm (*n* = 6). (G) Western blotting analysis of uncoupling protein‐1 (UCP1) protein content in SAT. Every lane contains one mouse sample. In statistical chart, every point represents one mouse sample in the three experimental replications (*n* = 6). (H) Relative mRNA levels of browning‐related genes in SAT (*n* = 6). Data are expressed as the mean ± standard deviation (SD). Data are analyzed by unpaired Student's *t*‐test (B, C, and F‒H) or two‐way analysis of variance (ANOVA) followed by Bonferroni's test (A, D, and E). ^*^ *p* < 0.05, ^**^ *p* < 0.01, ^***^ *p* < 0.001.

Activation of beige cells can restore whole‐body metabolic homeostasis and improve obesity‐related metabolic dysfunction. Hence, to further explore the pharmaceutical effects of NJT‐2 on metabolic disorders, we generated diet‐induced obesity in mice and treated them with NJT‐2 (10 mg/kg) daily for 2 weeks. As shown in Figure 6A, after 14 days of treatment, the mice treated with NJT‐2 showed a remarkable decrease in body weight gain. By manually evaluating adipose mass, we found that NJT‐2 significantly reduced the mass of SAT, but had limited effect on EAT and BAT (Figure 6B). Serum concentrations of triacylglycerols (TG), total cholesterol (T‐CHO), and low‐density lipoprotein cholesterol (LDL‐C) dramatically decreased upon NJT‐2 treatment (Figure 6C). Glucose‐tolerance test (GTT) and insulin‐tolerance test (ITT) indicated that NJT‐2 significantly improved insulin sensitivity (Figure 6D,E). We analyzed the histological features of the adipose tissues and the UCP1 protein content in SAT. The result in Figure 6F revealed that NJT‐2 markedly decreased the lipid droplet size in SAT, suggesting NJT‐2 promoted the lipid degradation. Meanwhile, the UCP1 protein was dramatically induced by NJT‐2 administration in SAT (Figure 6G). Subsequent mRNA quantitation of beige biomarkers in SAT confirmed that NJT‐2 strongly induced the expression of beige cell marker genes such as *Ucp1*, *Cidea*, *Dio2*, and *Pgc1a* (Figure 6H). In parallel, the evaluation of adipocytes in the EAT and BAT showed that NJT‐2 decreased the size of adipocytes (Figure S8A,B), suggesting that NJT‐2 was able to attenuate the lipid content in adipose tissues. At last, we evaluated the pathological features of the peripheral metabolic organs, including the liver and skeletal muscles. The serum levels of ALT, AST, and alkaline phosphatase (ALP) were dramatically decreased upon NJT‐2 treatment (Figure S8C), and NJT‐2 significantly attenuated steatosis of the liver and muscle tissues (Figure S8D,E). Considered together, these data confirm that NJT‐2 regulates adipose‐browning remodeling and can improve obesity‐related metabolic disorders.

### 2.7 NJT‐2 does not have the adverse effects seen with RSG

TZDs have been used as antidiabetic drugs and act as insulin sensitizers in the treatment of type 2 diabetes mellitus. However, the clinical use of TZDs is limited by their adverse effects, including cardiotoxicity, sodium and water retention, and osteoporosis. Here, to test whether NJT‐2 had the similar side effects of TZDs, we treated high‐fat diet‐induced obese mice with RSG (10 mg/kg) and NJT‐2 (10 mg/kg) for 14 days. After the treatment, we first compared pharmacological efficacy between NJT‐2 and RSG on metabolic improvement. As shown in Figure S9A, the levels of TG, T‐CHO, and LDL‐C in the plasma of mice treated with RSG and NJT‐2 significantly decreased, whereas we observed the TG level in the NJT‐2 group was lower than that in the RSG group. The GTT and ITT results showed that NJT‐2 possessed the similar insulin sensitizing effect to RSG (Figure S9B,C). Moreover, the effect of NJT‐2 in reducing lipid droplet size is stronger than that of RSG (Figure S9D). Together, these data indicated that NJT‐2 had the similar pharmacological efficacy to RSG in regulating metabolic disorder.

**FIGURE 7:**
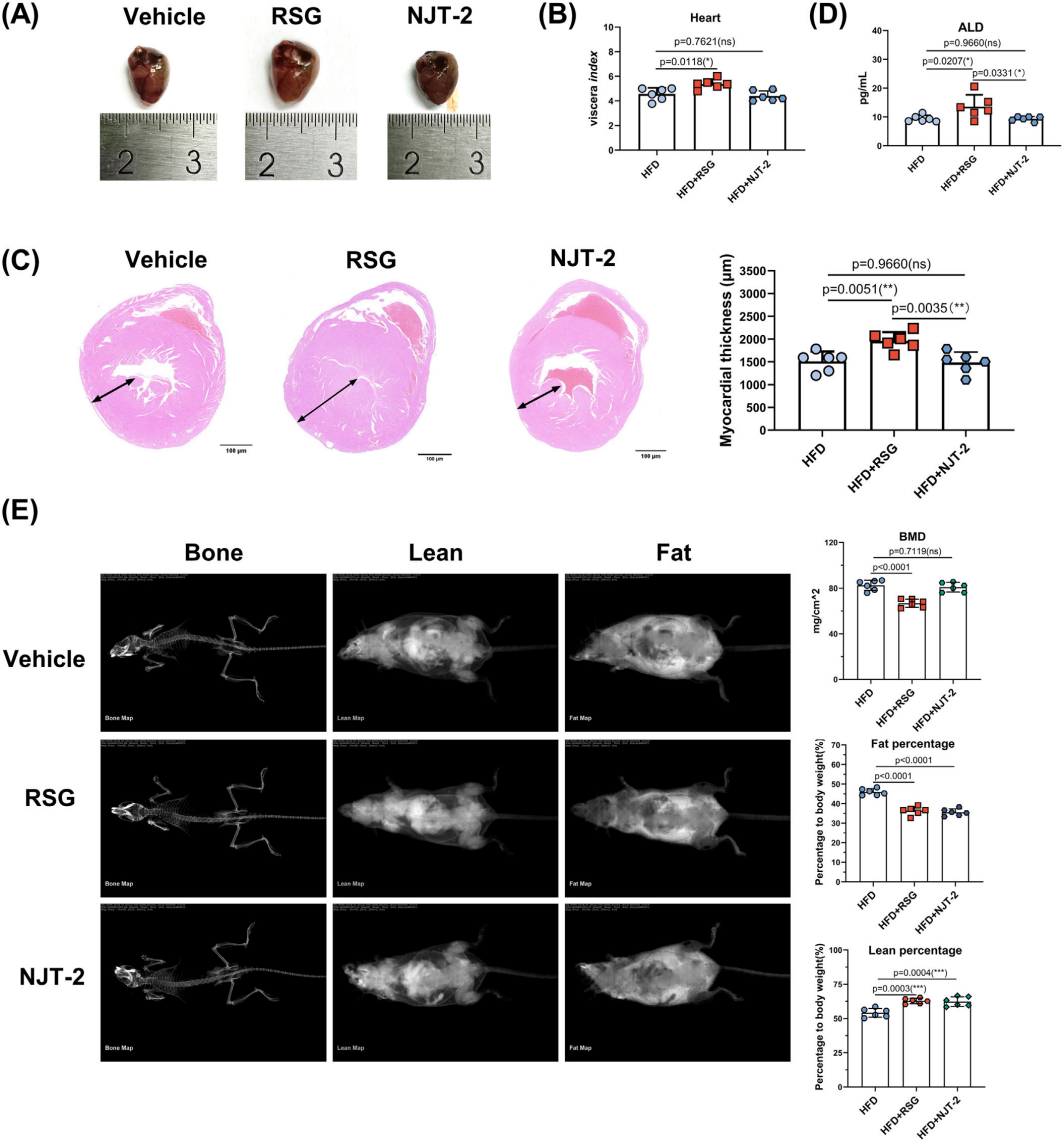
NJT‐2 does not have the adverse effects observed in rosiglitazone (RSG). (A and B) High‐fat diet (HFD) obese mice were treated with NJT‐2 or RSG (10 mg/kg) for 14 days. Hearts were isolated. The representative light field photo of hearts (A) and the viscera index of hearts (B) (*n* = 6). (C) Hematoxylin‒eosin (H&E) staining of hearts. 10× magnification, scale bar, 100 µm. The wall thickness of the left ventricle was analyzed by ImageJ (*n* = 6). (D) The plasma aldosterone (ALD) level was analyzed by commercial kit (*n* = 6). (E) Dual‐energy X‐ray absorptiometry (DEXA) scans showing lean/fat percentage and bone mineral content (BMC) upon NJT‐2 or RSG treatment (*n* = 6). Data are expressed as the mean ± standard deviation (SD). Data are analyzed by one‐way analysis of variance (ANOVA) followed by Dunnett's test (B and E) or one‐way ANOVA followed Tukey's test (D and C). ^*^ *p* < 0.05, ^**^ *p* < 0.01, ^***^ *p* < 0.001.

Next, the adverse effects that identified in RSG had been evaluated. The hearts from RSG treatment group exhibited significant increased viscera index than those in vehicle group. However, NJT‐2 treatment did not change the index (Figure 7A,B). Furthermore, the pathological section analysis indicated that RSG dramatically caused the myocardial hypertrophy (increase in the diameter of the wall thickness of the left ventricle), while NJT‐2 had no effect (Figure 7C). Thus, NJT‐2 did not induce the cardiotoxic side effect. Moreover, unlike RSG led to the increase of plasma aldosterone (ALD), a maker of water‒sodium retention, NJT‐2 treatment failed to enhance the ALD level (Figure 7D), hinting that NJT‐2 exhibited lower side effect on blood pressure stability and electrolyte balance. Osteoporosis is another side effect of RSG. We also analyzed the effect of NJT‐2 on bone content. Dual‐emission X‐ray absorptiometry evaluation showed that both RSG and NJT‐2 significantly reduced fat body mass and increased lean body mass. However, NJT‐2 did not induce decreased bone mineral content (a key marker of bone loss) (Figure 7E), which was significantly decreased by RSG treatment. Considered together, these results indicate that NJT‐2 is a safer modulator of PPARγ than RSG without the adverse effects.

## 3 DISCUSSION

PPARγ is a ligand‐dependent transcriptional factor, with a Y‐shape LBD to sense and bind the endogenous or exogenous ligands. In LBD, 12 α‐helix and four β‐sheets constitute a highly flexible hydrophobic core for ligand binding.^30^ In the absence of a ligand, the α‐helix exhibits dynamic conformations in a balance between the active and inactive states. Binding of ligands, such as TZDs, stabilizes the conformation of the LBD, especially helix H12 dynamics; changes the co‐factor binding surface; and finally recruits co‐activators to transactivate downstream gene expression.^31^ In contrast to complete agonists, SPPARγMs tend to destabilize helix H12, and their binding enhances the stabilization of helix H3 and the β‐sheet region in LBD.^1^, ^32^ For example, the partial agonist VSP‐77 forms an active conformation with its C‐terminal activation function‐2 helix (AF‐2), packed closely with helices 3 and 4 of the LBD. This binding mode does not induce excessive adipogenesis gene transcription but activates insulin‐sensitivity gene expression.^33^ MRL‐24 and SR1664 are positioned close to helix H3 and in contact distance with Glu343, Ile341, Leu340, and Met348, thereby not robustly stabilizing H12 but stabilizing the β‐sheet region of the LBD.^22^, ^32^ This is a milestone study because this is the first study to raise the notion that specific modulation of the PTM of PPARγ by SPPARγMs can control unique subsets of target genes. In our previous investigation, we showed that CDDO was the SPPARγMs for PPARγ T166 phosphorylation, thus dramatically promoting beige‐adipocyte differentiation^24^; however, the binding pocket and position of CDDO have not been studied in detail. In this study, we determined the detailed binding positions of CDDO and identified an uncharacterized binding pocket for small molecules. This pocket is positioned at the helix H3 and H2ʹ regions. In this binding region, C28 carboxyl of CDDO forms a hydrogen bond with Ser342, thereby stabilizing PPARγ.

As a partial agonist of PPARγ, the pharmacological functions of CDDO have been well studied in many disease models, such as inflammation, metabolic disorders, and cancers.^34^, ^35^, ^36^ However, this triterpenoid compound showed strong cytotoxic activity in normal cells.^37^ Considering these drawbacks, the development of specific, active, and less toxic compounds for binding to the novel pocket that we have obtained is the next emerging question. Therefore, we focused on herbs from TCMs that have been proven to possess the capacity to treat obesity,^38^, ^39^, ^40^ and the active natural products in these herbs were constructed as a database for screening. By performing 2D and 3D similarity‐based virtual screening, we successfully identified the novel SPPARγM—NJT‐2. NJT‐2, also called saikosaponin A, is a small natural compound derived from *Bupleurum chinense DC*, a TCM. Similar to CDDO, NJT‐2 formed hydrogen bonds with ARG‐280, GLU‐259, SER‐342, GLU‐291, and GLU‐295 residues in helices 3 and 2′. According to predicted docking‐score value, the binding affinity of NJT‐2 to PPARγ was stronger than that of CDDO, which was further verified by luciferase reporter system. Subsequent bioactivity evaluations indicated that the effects of NJT‐2 in promoting beiging and improving metabolic disorders were significantly greater than those of CDDO. The successful screening and identification of NJT‐2 not only demonstrates the role of this small molecule in promoting beige adipocyte generation, but also confirms that the NJT‐2 binding site is a screening pocket available for SPPARγM development. However, in contrast to the strong cytotoxicity of CDDO, NJT‐2 is milder and less toxic, and high doses do not cause significant damage to cells or animals. Moreover, compared with RSG, NJT‐2 does not exhibit the side effects associated with traditional PPARγ complete agonists, particularly in terms of cardiovascular toxicity and osteoporosis. Collectively, these findings indicate that NJT‐2 is a safe and effective metabolic regulator of SPPARγM.

Based on the efficacy results, the druggability of NJT‐2 should be considered in the context of translational medicine. First, the pharmacokinetics of NJT‐2 (saikosaponin A) is critical for its therapeutic outcome and safety. Several studies have reported the pharmacokinetics of NJT‐2 following oral or intravenous (IV) administration in rat models. NJT‐2 exhibited a moderate stability profile, with a plasma half‐life of approximately 4‒6 h.^41^, ^42^, ^43^ Under these conditions, the absorption kinetics of saikosaponin A is characterized by the time to reach maximum concentration (*T*_max_), typically within 1‒2 h post‐administration, indicating rapid absorption from the gastrointestinal tract. The peak plasma concentration (*C*_max_) observed in these studies suggests an effective passage through the intestinal barrier, which is essential for oral bioavailability. However, the reported bioavailability is relatively low, at approximately 23%, which may be due to extensive first‐pass metabolism in the liver.^41^ On IV administration, NJT‐2 is directly delivered into the bloodstream to ensure immediate and complete bioavailability. The distribution phase of NJT‐2 post‐IV injection is characterized by rapid distribution in tissues, reflected by a high initial volume of distribution. However, the clearance rate of NJT‐2 is moderately high. It is primarily metabolized by cytochrome P450 enzymes in the liver, which convert it into various metabolites. Metabolites of NJT‐2, such as saikosaponin D and C, have been identified in plasma samples, indicating a biotransformation process in the body.^44^ The elimination half‐life (*t*_1/2_) of NJT‐2 after IV administration is reported to be approximately 3‒5 h. Actually, many compounds from TCMs have disadvantages in terms of physicochemical properties, such as low bioavailability, short half‐life, and poor water solubility, which require further structural modification.^45^ For example, owing to the poor physicochemical properties of artemisinin, scientists have conducted extensive research on its structural modifications and synthesized dihydroartemisinin, artesunate, artemether, and other derivatives that have achieved good therapeutic effects against malaria.^46^, ^47^ Another new structural derivative, YY‐23, obtained by the structural modification of Zhimu saponin, showed significant antidepressant activity.^48^ Considering these findings, to further enhance the druggability of NJT‐2, future efforts should focus on structural modifications and optimization to obtain more stable and active derivatives. Additionally, novel drug‐delivery systems such as liposomes and nanoparticles can be designed for more efficient delivery of NJT‐2 into adipose tissues to achieve better browning effects. For instance, it has been reported that NJT‐2 was encapsulated by liposomes or other vehicles to improve its stability and solubility and bioavailability. The study showed that NJT‐2‐Lip had a prolonged circulation time, increased area under the curve and T1/2β for NJT‐2 after intravenous administration compared to solution.^49^ Another study reported that MePEG‐NJT‐2‐PCL exhibited more antiepileptic effects than NJT‐2 in an acute mouse epilepsy model.^50^

This study has some limitations. In drug research, the examination of potential biases, confounding factors, and technical challenges plays a crucial role in determining the validity and reliability of results. For example, the selection bias of animal models in the evaluation of beige‐adipocyte biogenesis may have influenced this conclusion. Although the scientific community typically utilizes the C57/BL6J mouse strain for studying adipocyte differentiation and function, research evidence suggests that this mouse strain is more sensitive to metabolic fluctuations and exhibits a stronger response to metabolism‐regulating drugs than other strains. Therefore, validation of the pharmacological functions in multiple mouse strains is necessary for pharmaceutical research. In the later stages of development, large animal models may be used to confirm the efficacy. Hence, in this study, we selected C57/BL6J mice solely for proof‐of‐concept validation, and in subsequent research, we will further analyze the efficacy of NJT‐2 in various mouse models of different strains. Moreover, confounding factors, such as animal age and gender, may also influence the conclusions. In this study, we chose male mice as the model. However, some studies have shown that adipose tissue in male mice is more prone to browning. Male mice are commonly used in the fields of metabolic pharmacology and adipose biology, and considering drug efficacy in both sexes simultaneously in drug research helps to further elucidate the effectiveness of drugs. Furthermore, technical challenges such as concerns with measurement techniques may have affected the interpretation of the results. In our study, the investigation of adipose tissue primarily relied on manual weighing and pathological examination. Manual weighing may introduce errors, leading to potential deviations in the statistical analysis of the adipose‐tissue content. Furthermore, owing to limitations in image capturing, the quantitative analysis of the morphology of the entire adipose tissue from pathological sections is challenging. In the future, we will utilize more advanced techniques, such as panoramic tissue quantification and nuclear magnetic resonance systems, to conduct a more detailed study on the browning effect of NJT‐2.

In summary, we successfully identified a novel ligand‐binding pocket in PPARγ LBD, and discovered NJT‐2 is a new SPPARgM. NJT‐2 showed greater activity than CDDO in inducing beige adipocyte differentiation and effectively improved HFD‐induced obesity. Our results provide advanced molecular insights into the structural details of ligand‐binding in PPARγ. Thus, the development of novel PPARγ ligands targeting the new pocket may provide a new therapeutic avenue for the treatment of metabolic diseases.

## 4 MATERIALS AND METHODS

### 4.1 Chemicals, reagents, and plasmids

Commercial chemicals and reagents, including NJT‐2 (IS0500, Solarbio), ginsenoside Rg3 (IG0260, Solarbio), CDDO (HY‐14909, MCE), RSG (HY‐17386, MCE), a dual‐luciferase reporter assay kit (E1910, Promega), a one‐step cloning kit (C112‐01, Vazyme), a reactive oxygen species assay kit (S0033S, Beyotime), and Griess reagent (S0024, Beyotime), were purchased. PPRE‐luc (E4121) and pRL (E2261) plasmids were purchased from Promega. The cDNA sequence of mouse PPARγ was cloned into the pLenti6/v5 lentiviral expression vector using the homologous recombination method. Site‐specific mutants of PPARγ were generated using the mut express II fast mutagenesis kit based on PCR (C214‐01, Vazyme).

### 4.2 Binding pose analysis

The potential pockets of PPARγ (PDB: 3E00) were predicted using the SiteMap of Maestro 11.5 (Schrodinger). The predicted pockets were refined as receptor grids for docking analysis. The binding pose analysis of the CDDO‒PPARγ complex was generated using the Glide of Maestro in accordance with the standard procedure. The docking results with the highest scores were visualized using PyMoL 2.5 (Schrodinger). The HDX‐MS data were reanalyzed according to our previous paper^6^ and visualized by PyMOL.

### 4.3 Molecular dynamics simulation

MD simulation of the CDDO‒PPARγ complex was performed using Gromacs 2020 (KTH Royal Institute of Technology). Briefly, the AMBER99SB‐ILDN force field was used for the protein, and the GAFF force field was used for the ligand. After cleaning, the solvation of complexes was performed in an SPC/E water model. NaCl (0.15 M) was added to neutralize the system. Energy minimization was performed for 5000 steps using the steepest descent method. The simulation time was set to 50 ns. After the simulation was complete, the RMSD tracks and occupancies of hydrogen bonds were calculated.

### 4.4 In‐house library preparation

The three ancient Chinese medical texts (Shang Han Lun, Jin Kui Yao Lve, and Pi Wei Lun) were reviewed and the formulas in these ancient texts that can treat the metabolic diseases were collected, including Banxia xiexin decoction, Sijunzi decoction, Lizhong decoction, Xiaojianzhong decoction, Buzhong Yiqi pill, etc. Then, 91 herbal medicines were identified in the formulas, including *Panax ginseng* C. A. Meyer., *Bupleurum chinensis* DC., *Glycyrrhiza uralensis* Fisch., *Pinellia ternata* (Thunb.) Breit., *Scutellaria baicalensis* Georgi, *Alisma plantago‐aquatica* Linn, *Magnolia officinalis Rehd*. et Wils, etc. Next, the online database: Next, TCMSP (http://tcmspw.com/tcmsp.php), TCMID (http://www.megabionet.org/tcmid/), and TCM and chemical components database of Shanghai Institute of Organic Chemistry (http://www.organchem.csdb.cn/scdb/default.asp), the three online databases were used to annotate the natural small molecular compounds in the 91 herbs. The total 4097 compounds were founded and recorded in the in‐house library.

### 4.5 2D fingerprint and 3D shape similarity

Two different similarity virtual screening strategies, 2D fingerprint and 3D shape similarity, were applied in parallel, using CDDO as a search query molecule (Figure 2A). All 4097 compounds were prepared using LigPrep with default parameters. We used Canvas 3.5 (Schrodinger) to generate three fingerprints for 2D virtual screening, including MACCS, ECFP, and topological. Compounds with a Tanimoto coefficient (*T*_c_) value of ≥0.6 were considered potential active compounds. 3D pharmacophore fingerprints were used for 3D shape similarity using Canvas. The top 20 ranked compounds were generated. After the removal of duplicates, absorption, distribution, metabolism, excretion (ADME) screening, and cluster analysis, 10 compounds were selected for further study.

### 4.6 Molecular docking and binding energy calculation

Molecular docking was performed using the Glide algorithm of Schrodinger to evaluate the ligand‒PPARγ interaction. The Ser342 residue was set as the receptor grid centroid in the structure of PPARγ. Ten compounds were analyzed using the standard precision method. The free energy of the binding (Δ*G* bind) for the ligand‒PPARγ complex, whose docked pose was retrieved from Glide, was determined using the Prime MM‐GBSA algorithm (Schrodinger).

### 4.7 PPARγ transactivation assays

The dual luciferase reporter assay was used to test PPARγ transactivation based on our experimental laboratory systems.^20^ Briefly, 5 × 10^4^ HEK 293T cells were seeded in 24‐well plates and allowed to reach 70%‒80% confluence. HEK 293T cells were transfected with mixed plasmids containing PPARγ, PPRE‐luc, and pRL using a Lipofectamine 2000 kit (11668019, Invitrogen) according to the manufacturer's protocol. After 24 h, luciferase activity was measured according to the modulus single tube operating manual (Promega). PPARγ transactivation activity was normalized to *Renilla* luciferase activity.

### 4.8 Microscale thermophoresis

The binding interaction between NJT‐2 and PPARγ‐LBD was detected by MST according to the procedure.^21^ In general, the method was involved in overexpression of the PPARγ‐GFP‐fused protein and cell lysis under non‐denaturing conditions. First, the cell lysate was prepared using RIPA buffer (P0013B, Beyotime) with protease inhibitors cocktail (HY‐K0010, MCE). After quantitative analysis by bicinchonininc acid (BCA), the protein was added into MST buffer (50 mM Tris HCl, 250 mM NaCl, 10 mM MgCl_2_, 0.05% Tween‐20, and 5% bovine serum albumin [BSA]). Finally, the protein mixtures (cell lysis and NJT‐2) were loaded into Monolith NT.115 series capillaries (NanoTemper), and analyzed by MO Control v1.6 software.

### 4.9 Primary adipocyte differentiation

ADSCs were isolated from the WAT of male C57BL/6J mice. First, adipose tissues were digested in Hank's buffer containing 5% BSA and 0.5 mg/mL collagenase type I and shaken at 150 rpm for 90 min at 37°C. Digested tissues were filtered through a 100‐µm strainer and centrifuged at 1500 rpm for 10 min. After washing with cold phosphate‐buffered saline (PBS) twice, the cells were cultured with Dulbecco's modified Eagle's medium (DMEM)/F12 medium containing 15% fetal bovine serum (FBS). After 48 h, the differentiation culture medium (5 µM dexamethasone, 850 nM insulin, 0.5 mM 3‐isobutyl‐1‐methylxanthine, 125 nM indomethacin, and 1 nM triiodothyronine) was replaced with the original culture medium to induce adipogenic differentiation. Mature adipocytes were harvested on differentiation day 8.

### 4.10 Oil red O and mitochondrial staining

We used oil red O staining (G1262, Solarbio) to analyze the content and distribution of lipid droplets in adipocytes. The cells were fixed in 4% paraformaldehyde for 20 min after washing with PBS three times. The cells were stained with oil red O for 2 h at 37°C. After washing five times for 2 min with PBS, the stained cells were photographed using fluorescence microscopy (Nikon). After discarding the PBS, 300 µL of dimethyl sulfoxide (DMSO) was added, and the absorbance value was measured at 570 nM for quantitative analysis.

The mitochondrial membrane potential of differentiated adipocytes was stained with MitoTracker Red (M22425, ThermoFisher) in serum‐free DMEM/F12 for 15 min at 37°C. The adipocytes were fixed with 4% paraformaldehyde for 15 min at 37°C after washing five times with PBS. After covering the anti‐fluorescence quenching sealing solution containing 4,6‐diamidino‐2‐phenylindole (DAPI) (C1005, Beyotime), LSM980 confocal microscopy (ZEISS) was used to analyze the membrane potential of the mitochondria.

### 4.11 Animal experiments

The male C57BL/6J mice (18‒20 g) were purchased from the Nanjing Biomedical Research Institute of Nanjing University (Nanjing) and housed in the experimental animal room of Nanjing University School of Life Science (Nanjing) with an specific pathogen free(SPF) grade. After 1 week of acclimatization, all mice were randomly assigned into three groups: the vehicles, CDDO, and NJT‐2 groups. Mice in the CDDO and NJT‐2 group were treated with CDDO (10 mg/kg) and NJT‐2 (10 mg/kg) for 2 weeks, respectively. Twelve hours after the last administration, all mice were anesthetized via intraperitoneal injection of tribromoethanol (200 mg/kg). The WAT, EAT, and BAT were collected for histopathological and qPCR analysis.

For obesity mouse model, the mice were induced by high fat diet (Xietong Pharmaceutical Bio‐Engineering). The assay kits of ALT (C009‐2‐1), AST (C010‐2‐1), ALP (C059‐2‐2), TG (C110‐1‐1), T‐CHO (C111‐1‐1), and LDL‐C (C113‐1‐1) were purchased from Nanjing Jiancheng Bioengineering Institute. The ELISA kit for ALD was purchased from Sangon Biotech (D751031).

### 4.12 Hematoxylin‒eosin staining and immunohistochemistry

For hematoxylin‒eosin (H&E) staining, we collected WAT, EAT, BAT, heart, liver, spleen, lung, kidney, and skeletal muscle from mice, fixed it immediately with 4% paraformaldehyde, and dehydrated in different ethanol concentrations before paraffin embedding. The tissues were cut into 5 µm thick slices and stained with H&E. After dewaxing, hydration, dyeing, dehydration, transparency, and sealing, samples were photographed using Pannoramic Scan (3DHISTECH).

For immunohistochemistry, WAT and BAT tissues were incubated with 5% BSA for 1 h then treated with a UCP1 primary antibody (1:500; ab209483, Abcam) at 4°C overnight. After washing three times with PBS, the tissue slices were incubated with a goat anti‐rabbit secondary antibody (1:1000; A0181, Beyotime) at room temperature (RT) for 1 h. The tissues were incubated with 3,3‐diaminobenzidine (DAB) and hematoxylin for 10 min. After dehydration with different concentrations of ethanol and dimethylbenzene, the tissues were covered with glass and dried at RT for half of the day. Each section was scanned using Pannoramic Scan.

### 4.13 The assay of CCK‐8, cell apoptosis, and TUNEL

The viability of cells cultured with the drugs was determined by the CCK‐8 (A311‐01, Vazyme). For the procedure, ADSC or 3T3‐L1 cells were cultured in 96‐well plates with 5 × 10^3^ cells per well. After 24 h, different concentrations of drug were added by changing the old medium, co‐cultured for 24 h in incubator. CCK‐8 reagent was added to each well at 10 µL per well, incubated for 3 h, then read at 450 nm using an Infinite 200 PRO microplate reader (TECAN) and the results recorded.

Apoptotic cells were measured using the Annexin V‐FITC/PI kit (A214‐01, Vazyme). According to the manufacturer's instructions, ADSC or 3T3‐L1 cells were resuspended in 100 µL binding buffer and stained with annexin V (5 µL) and PI (5 µL). Then, the cells were then incubated for 15 min in the dark and detected by the NovoCyte flow cytometry (Agilent).

For TUNEL assay, the mature adipocytes were fixed with 4% paraformaldehyde for 20 min. Next, the procedure was performed according to the TUNEL FITC Apoptosis Detection Kit (A111‐01, Vazyme). Finally, the stained cells were photographed using fluorescence microscopy (Nikon).

### 4.14 RNA‐seq and data analysis

Total RNA was extracted using Trizol reagent (15596026, ThermoFisher) following the manufacturer's instructions. Subsequently, next‐generation sequencing libraries were prepared according to the manufacturer's protocol and sequenced on a Novaseq 6000 (Illumina). The sequencing reads were aligned to the GRCm38 genome using the HISAT2 package. DESeq2 software was utilized for the analysis of differential gene expression between two distinct groups. Genes with a false discovery rate below 0.05 and an absolute FC >2 were considered differentially expressed genes. These genes were further investigated for enrichment of GO functions and KEGG pathways. The raw reads have been deposited in the NCBI Gene Expression Omnibus database with the accession number GSE267657.

### 4.15 Oxygen consumption assays

The OCR was measured using the XF24 Extracellular Flux Analyzer (Seahorse Biosciences). OCR values were derived from four independent measurements in this experiment. Baseline OCR was recorded every 10 min, before and after the sequential injection of oligomycin (1.5 µM), carbonyl cyanide‐4‐(trifluoromethoxy) phenylhydrazone (1 µM), and Rotenone and antimycin A (0.5 µM).

### 4.16 Glucose tolerance and insulin tolerance tests

For the GTT, mice were fasted for 12 h and then intraperitoneally injected with glucose (2 g/kg). In the ITT, mice were fasted for 12 h and intraperitoneally injected with insulin (0.6 U/kg). Blood samples were collected from the tail vein at specified time points (0, 15, 30, 60, 90, and 120 min) following the injection of either glucose (GTT) or insulin (ITT). Blood glucose levels were measured using a glucometer.

### 4.17 Cell culture

HEK293T was purchased from the Stem Cell Bank (Chinese Academy of Sciences), and cultured in DMEM supplemented with 10% FBS (BC‐SE‐FBS08, Sbjbio), 100 U/mL penicillin and 100 µg/mL streptomycin (60162ES76, Yesen). Cells were cultured in a humidified atmosphere with 5% CO_2_ at 37°C.

### 4.18 Quantitative real‐time PCR

Total RNA was isolated from adipocytes using TRIzol reagent (R401‐01, Vazyme), and cDNA was synthesized using a Hiscript III Reverse Transcriptase (R302‐01, Vazyme) kit. StepOnePlus (Applied Biosystems) was used for qPCR using AceQ qPCR SYBR Green Master Mix (Q111‐02, Vazyme). The sequences of the primers are shown in Table S3.

### 4.19 Western blotting

Proteins were extracted using whole cell lysis buffer containing protease and phosphatase inhibitors. After centrifugation at 4°C, the protein concentration was determined using a BCA assay kit (P0011, Beyotime). The extracted proteins were separated by electrophoresis on 10% sodium dodecyl sulfate polyacrylamide gel electrophoresis (SDS‐PAGE) gels and then transferred to polyvinylidene difluoride membranes (PB9220, Invitrogen) for immunoblotting. The primary antibodies against UCP1 (ab209483, Abcam), PPARγ (2443S, CST), PRDM16 (ab303534, Abcam), PGC1α (Abcam ab313559), and β‐actin (AF0003, Beyotime) were utilized.

### 4.20 Statistical analysis

All results are presented as means ± SD. Statistical analysis was performed with the two‐tailed Student's *t‐*test or one‐way analysis of variance followed by Dunnett's test or Tukey's test using Prism 9.0 (GraphPad). Dunnett's test was used to compare each mean to a vehicle mean, while Tukey's test was used to compare each pair. ^*^ *p* ≤ 0.05, ^**^ *p* ≤ 0.01, and ^***^ *p* ≤ 0.001 were considered statistically significant.

## AUTHOR CONTRIBUTIONS

Nanfei Yang, Pingping Shen, and Qiang Tian designed the study and analyzed the data. Qiang Tian, Miaohua Wang, Xueting Wang, Zhenli Lei, Dianhua Chen, and Wei Zheng performed the experiments. Owais Ahmad and Nanfei Yang provided scientific suggestions and contributed to the manuscript revision. Nanfei Yang supervised the project. Nanfei Yang, Pingping Shen, and Qiang Tian wrote and revised the manuscript. All authors have read and approved the final manuscript.

## CONFLICT OF INTEREST STATEMENT

The authors declare they have no conflicts of interest.

## ETHICS STATEMENT

All animal experiments were approved by the Institutional Animal Care and Use Committee of School of Life Sciences, Nanjing University (IACUC‐2210008).

## Supporting information

Supporting Information

Supporting Information

## Data Availability

The data included in this study are available upon request from the corresponding author. The raw reads have been deposited in the NCBI Gene Expression Omnibus database with the accession number GSE267657.
