# Supplementary material for: Identification of an alternative ligand‐binding pocket in peroxisome proliferator‐activated receptor gamma and its correlated selective agonist for promoting beige adipocyte differentiation
Source: MedComm (2020). 2024 Jul 10;5(7):e650. doi: 10.1002/mco2.650 (PMC11233932; doi:10.1002/mco2.650)
Supplement: Supplementary file 2 — Supporting Information [file MCO2-5-e650-s002.docx]

**Supplementary Information**

**Identification of an alternative ligand-binding pocket in peroxisome proliferator-activated receptor gamma and its correlated selective agonist for promoting beige adipocyte differentiation**

**Authors**

Qiang Tian1,2, Miaohua Wang1, Xueting Wang1, Zhenli Lei3, Owais Ahmad1, Dianhua Chen1, Wei Zheng1, Pingping Shen1,2*, Nanfei Yang1,2,3*

**Affiliations**

1 State Key Laboratory of Pharmaceutical Biotechnology and Department of Urology, The Affiliated Nanjing Drum Tower Hospital, The Affiliated Hospital of Nanjing University Medical School, School of Life Sciences, Nanjing University, Nanjing, 210023, China.

2 Shenzhen Research Institute of Nanjing University, Shenzhen 518000, China.

3 School of Pharmaceutical Sciences, Wenzhou Medical University, Wenzhou, Zhejiang 325035, China.

***Correspondence to:**

Nanfei Yang: nfyang@smail.nju.edu.cn (Lead Contact)

Pingping Shen: ppshen@nju.edu.cn

**Table S1-The characteristics of five pockets predicted by SiteMap**

| Pocket | Sitescore | Size | Dscore | Volume | Docking score |
| --- | --- | --- | --- | --- | --- |
| 1 | 1.072 | 508 | 1.084 | 1115.972 | -4.196 |
| 2 | 0.707 | 35 | 0.701 | 81.934 | -2.143 |
| 3 | 0.780 | 34 | 0.760 | 104.272 | -2.650 |
| 4 | 0.630 | 36 | 0.477 | 65.856 | -2.531 |
| 5 | 0.797 | 29 | 0.809 | 85.064 | -2.276 |

**Table S2-The characteristics of 10 compounds by similarity-based screening**

| **Compd. ID** | **Name** | **Structure** | **Formula** | **MW** | **LogP** | **Docking score** |
| --- | --- | --- | --- | --- | --- | --- |
| NJT-1 | Saikosaponin D | 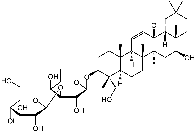 | C_42_H_68_O_13_ | 780.466 | 1.07 | 5.89 |
| NJT-2 | Saikosaponin A | 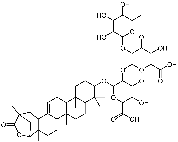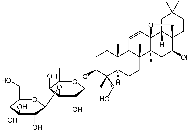 | C_42_H_68_O_13_ | 780.466 | 1 | 5.71 |
| NJT-3 | Licorice-saponin F3 |  | C_48_H_72_O_19_ | 952.4668 | 0.94 | 5.49 |
| NJT-4 | Saikosaponin B3 | 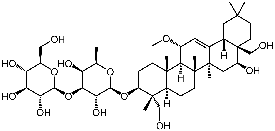 | C_43_H_72_O_14_ | 812.4922 | 0.16 | 4.96 |
| NJT-5 | Ginsenoside Rg3 | 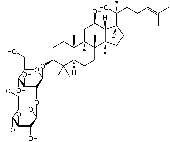 | C_42_H_72_O_13_ | 784.4973 | 1.53 | 4.53 |
| NJT-6 | Longispinogenin 3-O-beta-D-glucuronopyranoside | 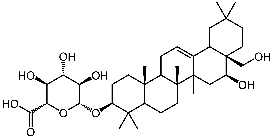 | C_36_H_58_O_9_ | 634.4081 | 3.36 | 4.18 |
| NJT-7 | Uralsaponin B | 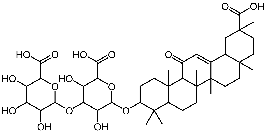 | C_42_H_62_O_16_ | 822.4038 | 1.89 | 4.12 |
| NJT-8 | 26-Deoxyactein | 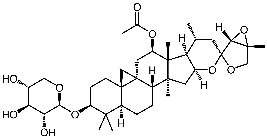 | C_37_H_56_O_10_ | 660.3873 | 3.98 | 3.11 |
| NJT-9 | Arjungenin | 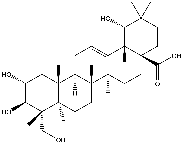 | C_30_H_48_O_6_ | 504.3451 | 3.45 | —— |
| NJT-10 | Hederagenin 3-O-arabinoside | 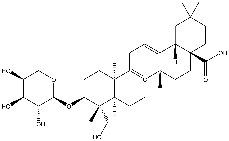 | C_35_H_56_O_8_ | 604.3975 | 1.22 | —— |

**Table S3-The primer sequence of genes for qPCR**

| **No.** | **Gene** | **Forward Primer** | **Reverse Primer** |
| --- | --- | --- | --- |
| 1 | 36B4 | AGATTCGGGATATGCTGTTGGC | TCGGGTCCTAGACCAGTGTTC |
| 2 | GAPDH | AGGTCGGTGTGAACGGATTTG | TGTAGACCATGTAGTTGAGGTCA |
| 3 | UCP-1 | AGGCTTCCAGTACCATTAGGT | CTGAGTGAGGCAAAGCTGATTT |
| 4 | PRDM16 | CCAAGGCAAGGGCGAAGAA | AGTCTGGTGGGATTGGAATGT |
| 5 | ACOT11 | AGGGGCTTCGCCTCTATGTT | TCCGGTATCCTTCACCCTCTG |
| 6 | COX7a | GCTCTGGTCCGGTCTTTTAGC | GTACTGGGAGGTCATTGTCGG |
| 7 | COX8b | TGTGGGGATCTCAGCCATAGT | AGTGGGCTAAGACCCATCCTG |
| 8 | ATGL | GGATGGCGGCATTTCAGACA | CAAAGGGTTGGGTTGGTTCAG |
| 9 | CIDEA | TGACATTCATGGGATTGCAGAC | GGCCAGTTGTGATGACTAAGAC |
| 10 | PGC1a | TATGGAGTGACATAGAGTGTGCT | CCACTTCAATCCACCCAGAAAG |
| 11 | ACOX | TAACTTCCTCACTCGAAGCCA | AGTTCCATGACCCATCTCTGTC |
| 12 | CPT1a | CTCCGCCTGAGCCATGAAG | CACCAGTGATGATGCCATTCT |
| 13 | PPAR-α | AGAGCCCCATCTGTCCTCTC | ACTGGTAGTCTGCAAAACCAAA |
| 14 | PPAR-γ | TCGCTGATGCACTGCCTATG | GAGAGGTCCACAGAGCTGATT |
| 15 | DIO2 | AATTATGCCTCGGAGAAGACCG | GGCAGTTGCCTAGTGAAAGGT |

**Supplemental Figures**


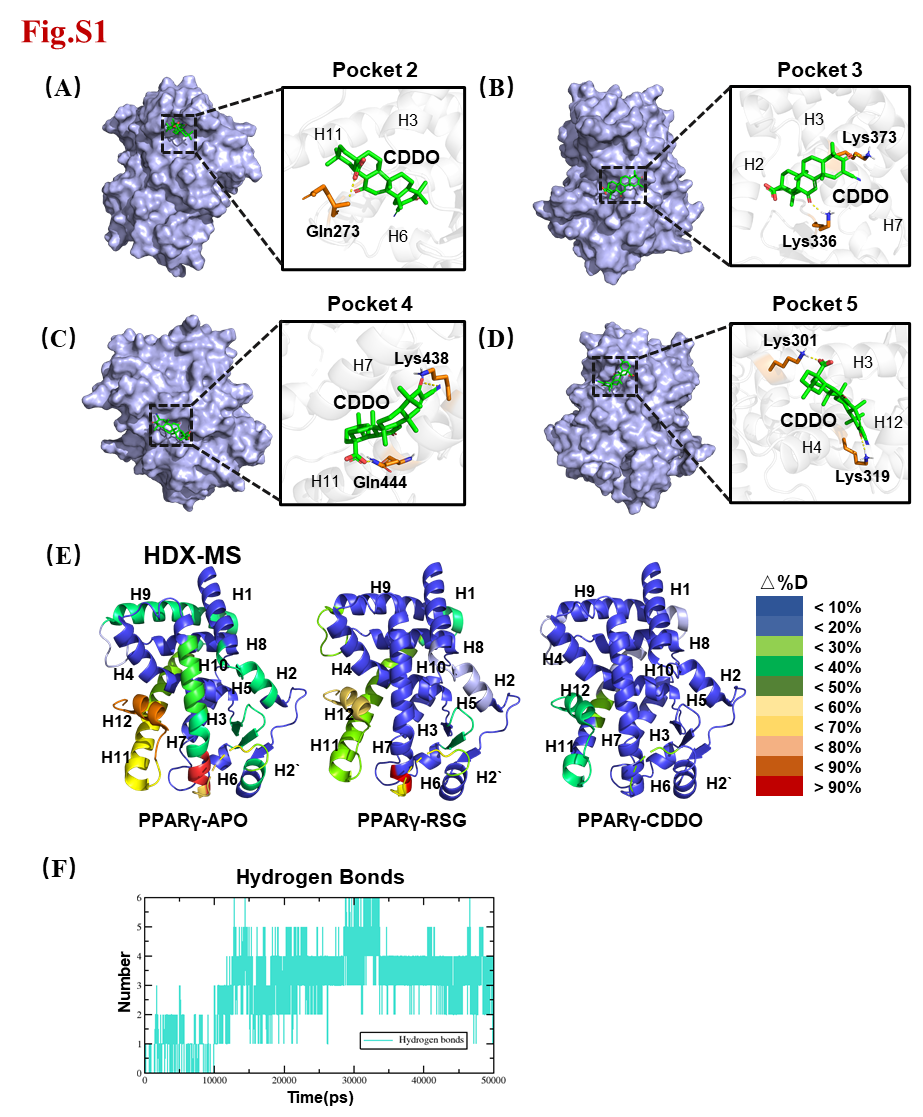


**Figure S1. Mapping the potential CDDO binding pocket in PPARγ.**

**(A-D)** The predicted pocket 2-4 that CDDO binding and the related interacted residues.

**(E)** HDX exchange rate mapping in PPARγ LBD (PDB:3e00). Percentages of deuterium difference are color-coded according to the color gradient key.

**(F)** Analysis of hydrogen bond occupancy using MD simulation.


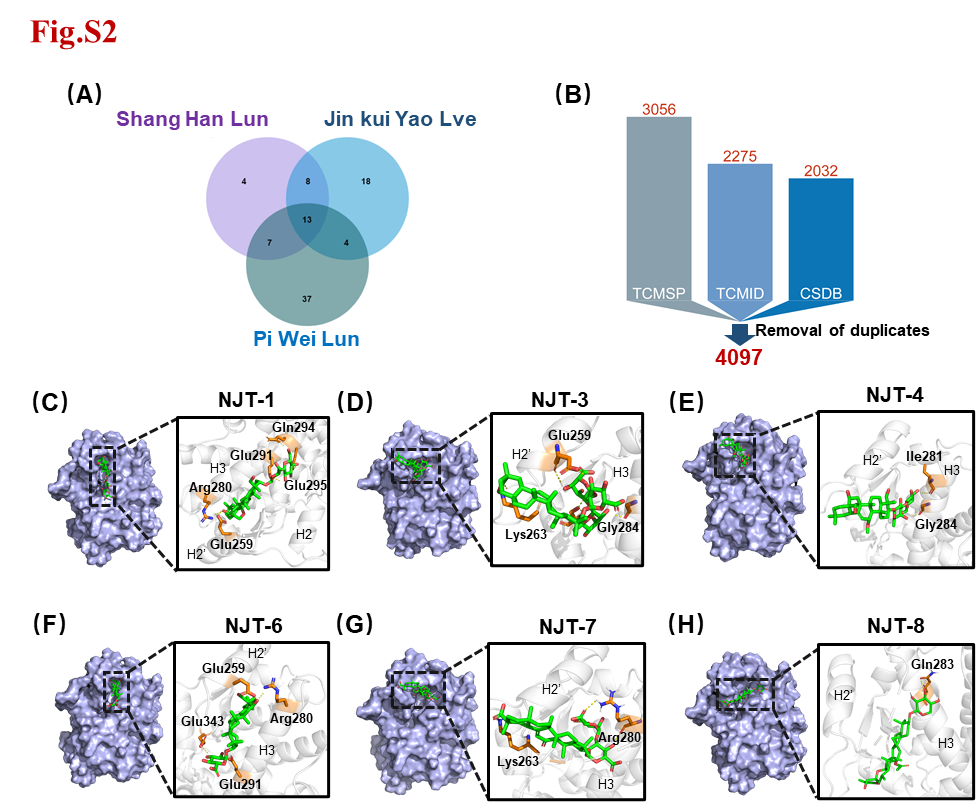


**Figure S2. In-house natural compound library construction and binding model predictions.**

**(A)** Ninety-one herbal medicines recorded in ancient Chinese medical texts (including Shang Han Lun, Jin Kui Yao Lve and Pi Wei Lun) were picked into library construction.

**(B)** TCMSP, YCMID, and CSCB online databases annotated 4097 natural compounds in the 91 herbal medicines.

**(C-H)** The docking model of NJT-1 (C), NJT-3 (D), NJT-4 (E), NJT-6 (F), NJT-7 (G) or NJT-8 (H) with pocket 1 in PPARγ LBD.


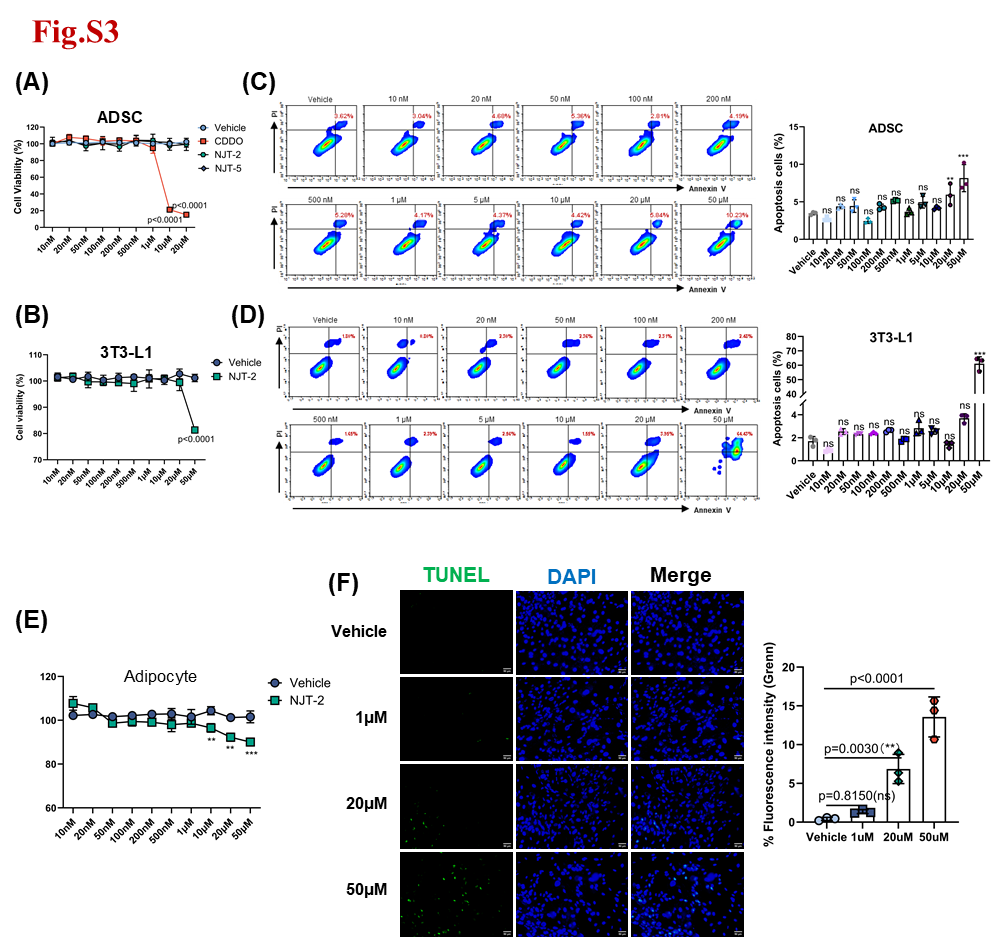


**Figure S3. NJT-2 cytotoxicity evaluation in ADSC, 3T3-L1 and mature adipocytes.**

**(A)** ADSCs were treated with CDDO, NJT-2 or NJT-5 for 24 h followed by the CCK8 test (n=3).

**(B)** 3T3-L1 cells were treated with NJT-2 for 24 h followed by the CCK8 test (n=3).

**(C-D)** Annexin V/PI combined with flow cytometry analysis of the effect of NJT-2 on ADSC (C) or 3T3-L1 (D) cell apoptosis. In statistical chart, every point represented one experimental replication (n=3).

**(E)** ADSCs were first differentiated into mature adipocyte and then the mature adipocytes were treated NJT-2 followed by the CCK8 test (n=3).

**(F)** TUNEL assay was performed to analysis the cytotoxicity of NJT-2 on mature adipocytes. In statistical chart, every point represented one experimental replication (n=3).

Data are expressed as the mean ± SD. Data were analyzed by one-way ANOVA followed by Dunnett's test (C-D and F) or two-way ANOVA followed by Bonferroni’s test (A-B and E). **^*^***P* < 0.05. **^**^***P* < 0.01, **^***^***P* < 0.001.


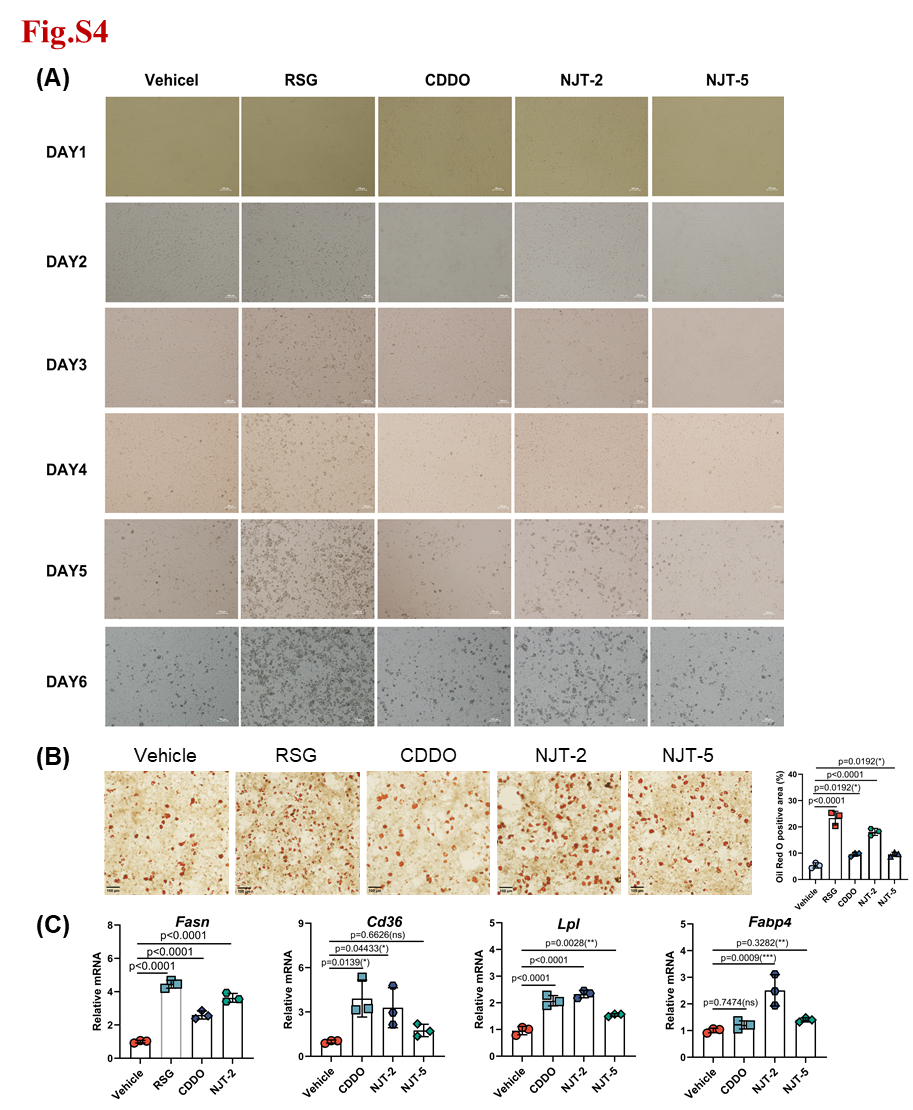


**Figure S4. NJT-2 and NJT-5 can promote the adipogenesis *in vitro*.**

**(A)** The ADSC cells were isolated from SAT and were differentiated into mature adipocytes with or without the compounds treatment. The compounds (RSG, CDDO, NJT-2 and NJT-5) concentration was 200 nM.

**(B)** Oil red O staining and quantitative analysis of the content of lipid droplets. In statistical chart, every point represented one experimental replication (n=3).

**(C)** Q-PCR analysis of adipogenic genes (n=3).

Data are expressed as the mean ± SD. Data were analyzed by one-way ANOVA followed by Dunnett's test (B-C). **^*^***P* < 0.05. **^**^***P* < 0.01, **^***^***P* < 0.001.


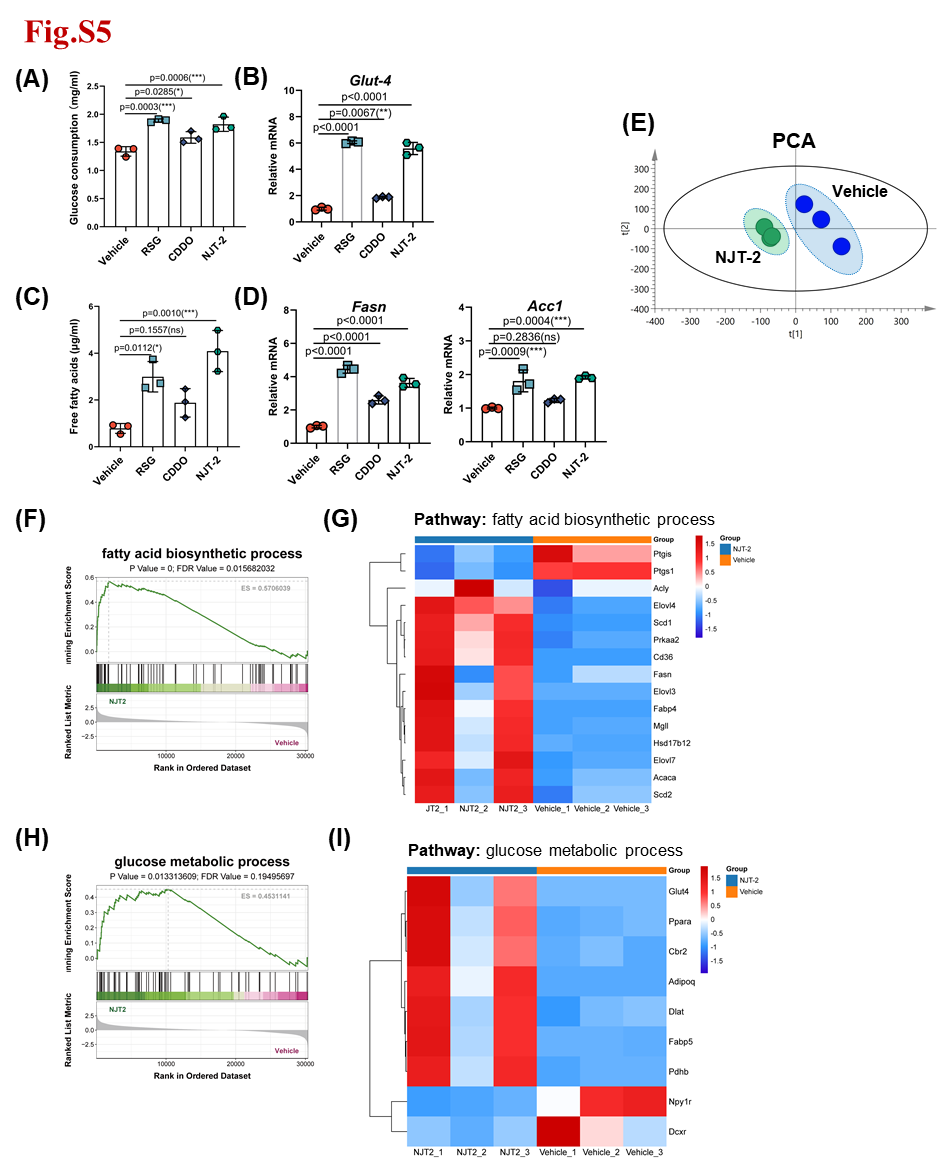


**Figure S5. NJT-2 reprograms the glucose and lipid metabolism in adipocytes.**

**(A)** The ADSC cells were isolated from SAT and were differentiated into mature adipocytes followed by compounds treatment. The compounds (RSG, CDDO and NJT-2) concentration was 200 nM. After the 24 h treatment, the glucose content was analyzed by commercial kit and the glucose consumption was calculated. In statistical chart, every point represented one experimental replication (n=3).

**(B)** related to (A), the *Glut4* gene expression was analyzed by Q-PCR (n=3).

**(C-D)** The fatty acid content in adipocytes was analyzed commercial kit (C) and the fatty acid synthetic genes were analyzed by Q-PCR (n=3).

**(E)** Principal component analysis (PCA) of differentially expressed genes in the RNA-Seq (n=3).

**(F-G)** GSEA analysis of fatty acid biosynthesis process (F) and the related gene expressions in this process (G) (n=3).

**(H-I)** GSEA analysis of glucose metabolic process (H) and the related gene expressions in this process (I) (n=3).

Data are expressed as the mean ± SD. Data were analyzed by one-way ANOVA followed by Dunnett's test (A-D). **^*^***P* < 0.05. **^**^***P* < 0.01, **^***^***P* < 0.001.


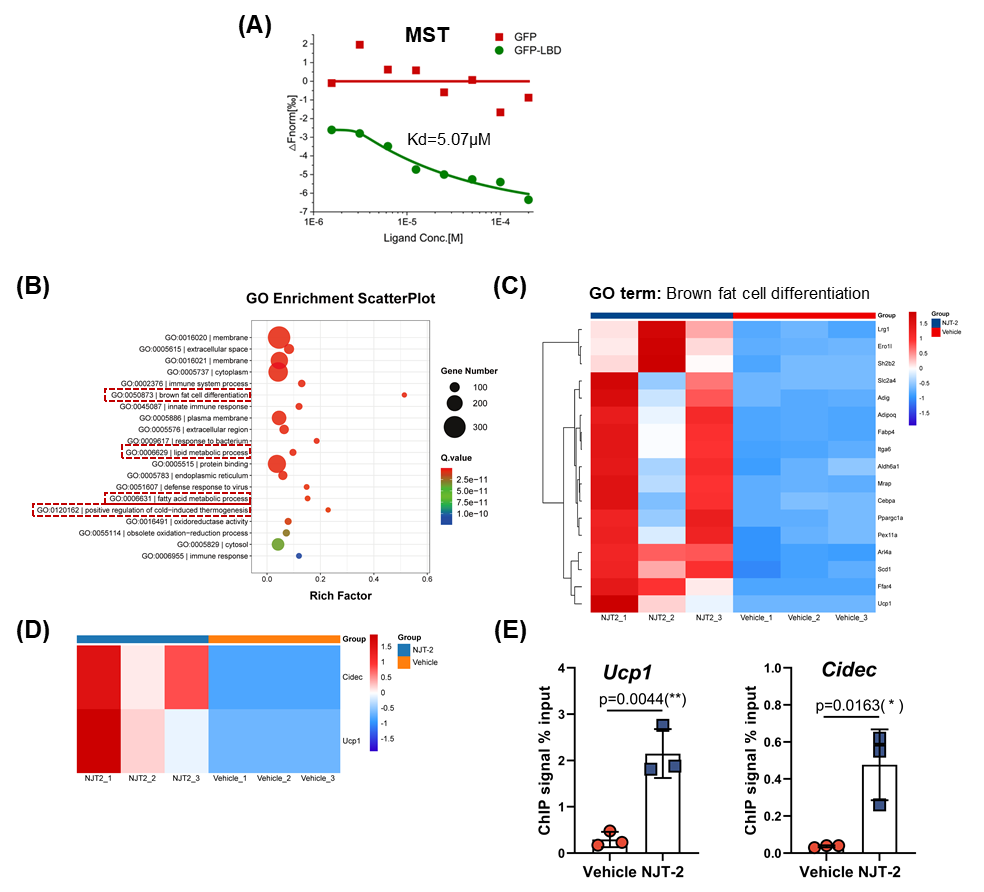


**Figure S6. NJT-2 promotes the PPARγ mediated beige cell gene transcriptions.**

**(A)** MST assay tested the interaction of NJT-2 to PPARγ LBD in 293T cells.

**(B)** GO analysis of differentially expressed genes between Vehicle and NJT-2 group (n=3).

**(C)** Heat map of differentially expressed genes related to brown fat cell differentiation process (n=3).

**(D)** Heat map of PPARγ targeted *Ucp1* and *Cidec* expressions (n=3).

**(E)** ChIP-qPCR analysis of PPARγ binding to the promoters of *Ucp1* and *Cidec* (n=3).

Data are expressed as the mean ± SD. Data were analyzed by unpaired Student’s t-test (E). **^*^***P* < 0.05. **^**^***P* < 0.01, **^***^***P* < 0.001.


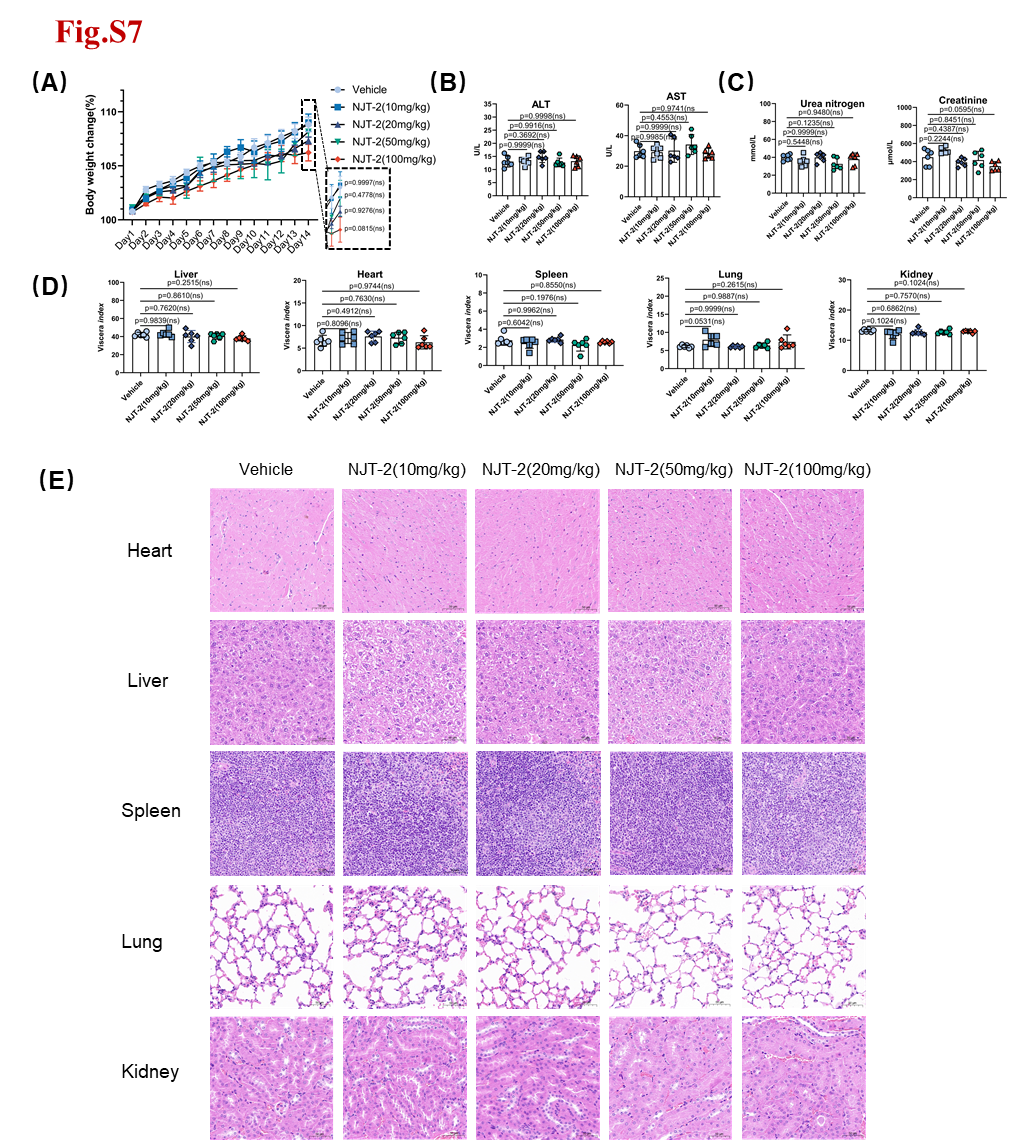


**Figure S7. Analysis of drug safety of NJT-2 in animal model.**

**(A)** Body weight curve of 6-week-old C57/B6J mice treated with (10, 20, 50, 100 mg/kg) for 14 days (n=6).

**(B)** The activities of ALT and AST in serum were measured by ALT/AST assay kits (n = 6).

**(C)** The level of urea nitrogen and creatinine in serum were measured by commercial kits (n = 6).

**(D)** The viscera index of key organs (percentage to body weight) (n = 6).

**(E)** H&E staining of key organs.

Data are expressed as the mean ± SD. Data were analyzed by one-way ANOVA followed by Dunnett's test (B-D) or two-way ANOVA followed by Bonferroni’s test (A). **^*^***P* < 0.05. **^**^***P* < 0.01, **^***^***P* < 0.001.


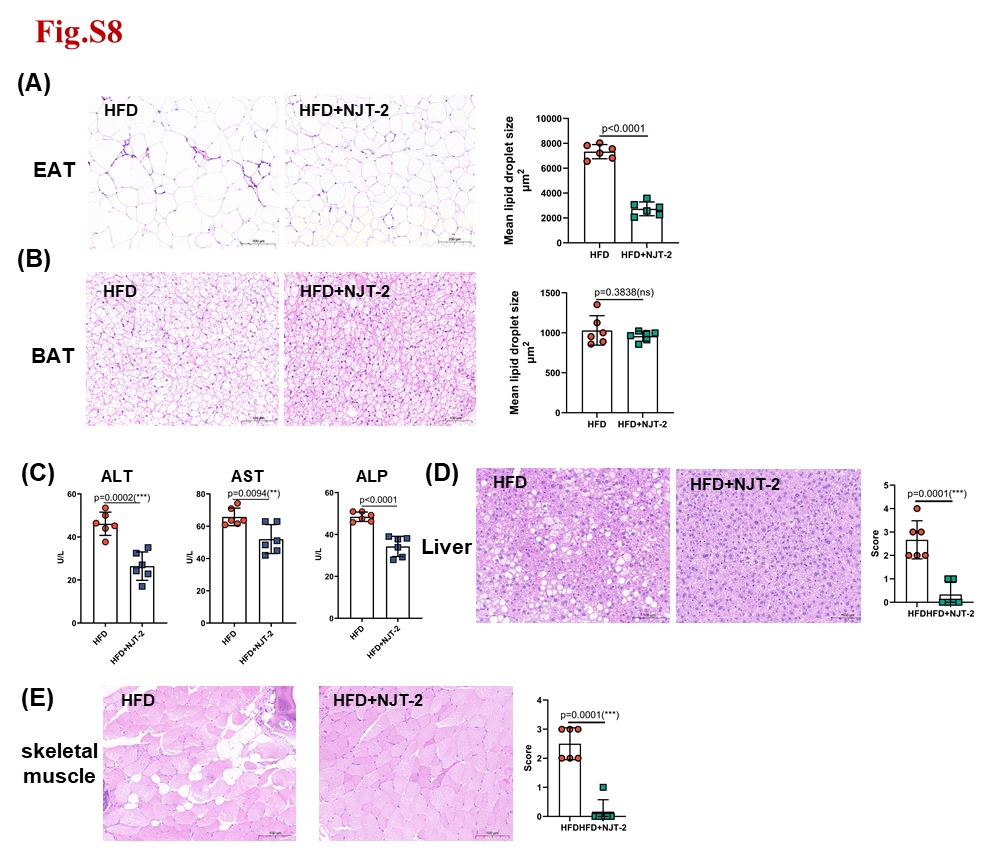


**Figure S8. NJT-2 improves the metabolic dysfunctions in adipose, liver and muscle.**

**(A-B)** H&E staining of EAT (A) and BAT (B). 100× magnification, scale bar, 100 μm. The mean lipid droplet size was analyzed by image J (n=6).

**(C)** The activities of ALT, AST and ALP in serum were measured by ALT/AST assay kits (n = 6).

**(D-E)** H&E staining and the pathological analysis of liver, and skeletal muscle. 100× magnification, scale bar, 100 μm (n=6).

Data are expressed as the mean ± SD. Data were analyzed by unpaired Student’s t-test (A-E) **^*^***P* < 0.05. **^**^***P* < 0.01, **^***^***P* < 0.001.


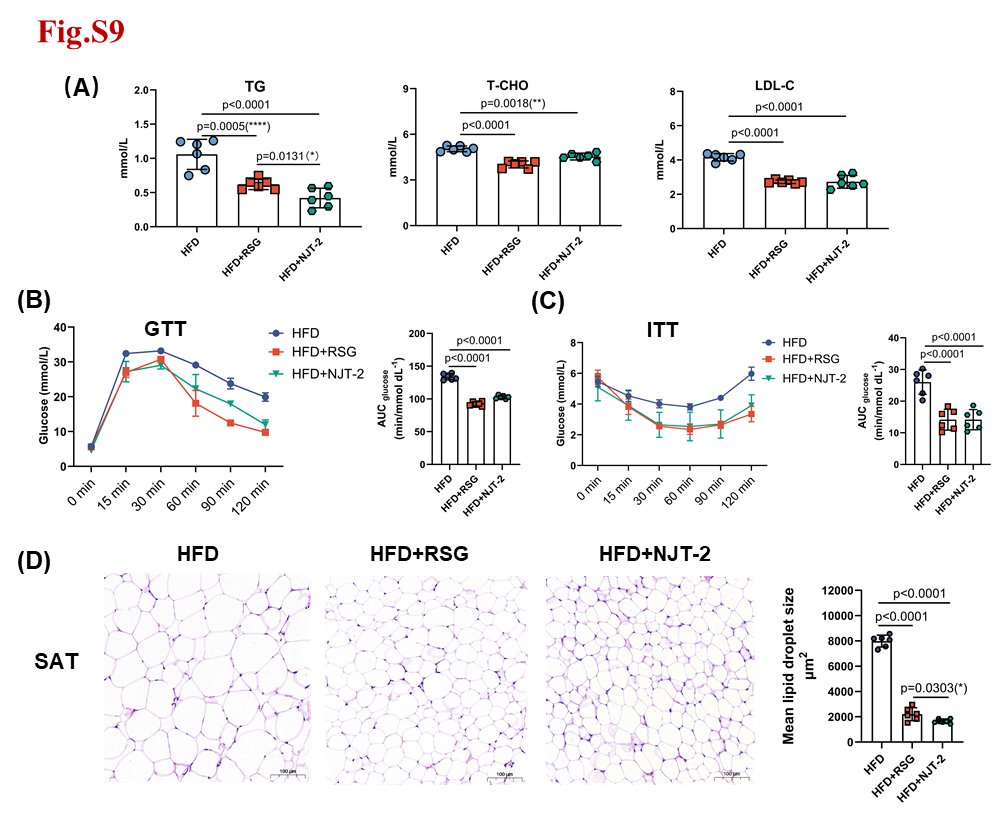


**Figure S9. NJT-2 exhibits similar metabolic improvement effect to RSG.**

**(A)** HFD obese mice were treated with NJT-2 or RSG (10 mg/kg) for 14 days. The concentrations of triacylglycerols (TG), total cholesterol (T-CHO) and Low-Density Lipoprotein Cholesterol (LDL-C) were measured by commercial kits (n = 6).

**(B-C)** Glucose tolerance test (GTT) (D) and insulin tolerance test (ITT) (E). The AUC was calculated and analyzed by Graphpad Prim software (n=6).

**(D)** H&E staining of SAT. 100× magnification, scale bar, 100 μm. The mean lipid droplet size was analyzed by image J (n=6).

Data are expressed as the mean ± SD. Data were analyzed by one-way ANOVA followed by Dunnett's test (B-D) or one-way ANOVA followed Tukey’s test (A)**^*^***P* < 0.05. **^**^***P* < 0.01, **^***^***P* < 0.001.
